# Human placental mesenchymal stromal cells are ciliated and their ciliation is compromised in preeclampsia

**DOI:** 10.1186/s12916-021-02203-1

**Published:** 2022-01-27

**Authors:** Sophia Indira Romberg, Nina-Naomi Kreis, Alexandra Friemel, Susanne Roth, Alice Steglich Souto, Samira Catharina Hoock, Kyra Fischer, Thorsten Nowak, Christine Solbach, Frank Louwen, Andreas Ritter, Juping Yuan

**Affiliations:** 1 Division of Obstetrics and Prenatal Medicine, Department of Gynecology and Obstetrics, University Hospital Frankfurt, J. W. Goethe- University, Theodor-Stern-Kai 7, D-60590 Frankfurt, Germany; 2 Medical practice for Gynecology, Mainzer Landstraße 265, D-60326 Frankfurt, Germany

**Keywords:** Primary cilium, Human chorionic villi mesenchymal stromal cells, Preeclampsia, Placental organoids, Motility, Cellular network formation

## Abstract

**Background:**

The development of the human placenta is tightly coordinated by a multitude of placental cell types, including human chorionic villi mesenchymal stromal cells (hCV-MSCs). Defective hCV-MSCs have been reported in preeclampsia (PE), a gestational hypertensive disease characterized by maternal endothelial dysfunction and systemic inflammation. Our goal was to determine whether hCV-MSCs are ciliated and whether altered ciliation is responsible for defective hCV-MSCs in preeclamptic placentas, as the primary cilium is a hub for signal transduction, which is important for various cellular activities.

**Methods:**

In the present work, we collected placental tissues from different gestational stages and we isolated hCV-MSCs from 1st trimester, term control, and preeclamptic placentas. We studied their ciliation, functionality, and impact on trophoblastic cell lines and organoids formed from human trophoblast stem cells (hTSCs) and from the trophoblastic cell line JEG-3 with various cellular and molecular methods, including immunofluorescence staining, gene analysis, spheroid/organoid formation, motility, and cellular network formation assay. The statistical evaluation was performed using a Student’s *t* test (two-tailed and paired or homoscedastic) or an unpaired Mann–Whitney *U* test (two-tailed).

**Results:**

The results show that primary cilia appeared abundantly in normal hCV-MSCs, especially in the early development of the placenta. Compared to control hCV-MSCs, the primary cilia were truncated, and there were fewer ciliated hCV-MSCs derived from preeclamptic placentas with impaired hedgehog signaling. Primary cilia are necessary for hCV-MSCs’ proper signal transduction, motility, homing, and differentiation, which are impaired in preeclamptic hCV-MSCs. Moreover, hCV-MSCs derived from preeclamptic placentas are significantly less capable of promoting growth and differentiation of placental organoids, as well as cellular network formation.

**Conclusions:**

These data suggest that the primary cilium is required for the functionality of hCV-MSCs and primary cilia are impaired in hCV-MSCs from preeclamptic placentas.

**Supplementary Information:**

The online version contains supplementary material available at 10.1186/s12916-021-02203-1.

## Background

### What is new?

This work shows that villous stromal cells including hCV-MSCs are ciliated ex vivo as well as in vitro. The primary cilium on these cells is dynamically regulated during gestation. PE placentas are associated with shortened ciliated hCV-MSCs. These hCV-MSCs are dysfunctional in their own functionalites as well as their interaction with trophoblastic cells. These data suggest that primary cilia are pivotal for functional hCV-MSCs possibly related to the placental development. hCV-MSCs with defective cilia might be connected with the progression of PE.

### What is relevant?

The placenta plays an essential role in supporting the development of the fetus. It represents an important reservoir of stem cells and transient progenitors including hCV-MSCs. Emerging evidence demonstrates that hCV-MSCs have multiple key roles in generating a functional microenvironment critical to a successful pregnancy. We show here that hCV-MSCs are ciliated and PE associated factors compromise primary cilia on hCV-MSCs and render them dysfuntional.

### Summary

Our data underscore the significant role of the primary cilium in the functional regulation of hCV-MSCs, which could be important for the development of the human placenta and might be associated with the pathogenesis of preeclampsia.

## Background

The human placenta is a complex transient organ and its development is strictly orchestrated by multiple signaling pathways and a variety of different placental cell types [1, 2]. Human placental villous cytotrophoblasts (hVCTBs) are able to differentiate into the human syncytiotrophoblast (hSTB) or extravillous trophoblasts (hEVTs) [2, 3]. Other cell types, such as Hofbauer cells, fibroblasts, vascular endothelial cells, and human chorionic villi mesenchymal stem/stromal cells (hCV-MSCs) are required for vascular engineering, angiogenesis, immunomodulation, and tissue homeostasis [4, 5].

MSCs are well known for their supportive functions in numerous biological processes by modulating the inflammatory and immune response, angiogenesis, and recruiting tissue-specific progenitor cells to sites of injuries [6]. Human placental MSCs have been isolated from different regions of the placenta, such as human basal decidua mesenchymal stromal cells (hBD-MSCs) [7], human chorionic mesenchymal stromal cells (hCMSCs) [8], human chorionic villi mesenchymal stromal cells (hCV-MSCs) [9], chorionic cotyledons, intervillous space mesenchymal stromal cells (CIV-MSCs) [10], and human chorionic plate mesenchymal stromal cells (hCP-MSCs) [11]. Accumulating evidence indicates that hCV-MSCs are of key importance in generating a functional microenvironment, particularly within diverse villus types of the human placenta. They are also critical for successful pregnancies and defective in placenta-related diseases like preeclampsia (PE) [12]. However, it is not well understood how these cells are regulated during placental development and why they are defective in preeclamptic placentas.

The primary cilium is a microtubule-based structure protruding from the cell surface. It acts as a signaling hub for a variety of molecular pathways between the cell and the extracellular environment through multiple receptors on its surface [13]. A pathway exclusively mediated by the primary cilium is the sonic hedgehog (Hh) pathway [14], which is of fundamental importance in the early embryogenesis, and its deregulation leads to a variety of severe genetic diseases, conserved across multiple organisms like humans, mice, and zebrafish [15, 16]. Recently, we reported that PE has a significant impact on the functionality of the primary cilium in trophoblasts [17].

PE is a pregnancy-related hypertensive disease with an estimated incidence rate of 2–8% of all pregnancies worldwide [18]. It is characterized by impaired placentation [19], and deregulated innate and adaptive immune system [20], and associated with increased vascular resistance and vasoconstriction leading to maternal hypertension [21]. These pathological cellular processes include reduced hVCTB proliferation, decreased hSTB fusion [22], impaired EVT invasion, an unbalanced immune response, and a disturbed differentiation capacity of trophoblastic cells [23, 24]. Despite decades of research, its molecular mechanisms are still not completely defined.

In the present work, the results indicate that human placenta MSCs, in particular hCV-MSCs, are ciliated throughout various stages of pregnancy. Moreover, hCV-MSCs with functional cilia are able to differentiate into multiple lineages and promote cellular network formation, as well as the motility and migration of trophoblastic cells, and the growth and function of human placental organoids. Importantly, hCV-MSCs derived from PE placentas displayed truncated primary cilia. Defective cilia compromised the function of hCV-MSCs, which might be connected to the pathogenesis of PE.

## Methods

### Human placental tissue samples

Following human placental tissue samples were used in this work: (1) formalin-fixed and paraffin-embedded 1st trimester placental tissue sections (*n* = 6) for examining primary cilia were kindly provided by Dr. Qi Chen, University of Auckland, New Zealand, and Fudan University, Shanghai, China. The collection of these samples was approved by the Ethics Committee of the Hospital of Obstetrics and Gynecology of Fudan University, China (reference number: 2018-62). The patients’ age was 20 to 33 years and gestational age was 6 to 9 weeks. (2) Second trimester (*n* = 4), early third trimester (*n* = 6) and late third trimester (*n* = 5) formalin-fixed and paraffin-embedded placental tissues were used for examining primary cilia. The donor clinical conditions leading to the delivery in the second and early third trimester are described in the supplement information (point 1.10). Further clinical information of these 15 donors is listed in Additional file 5 (Tab. S1). (3) Comparison of the cilium length and the percentage between matched controls and PE groups were performed with early third trimester placental tissues (*n* = 8) from women undergoing premature birth due to other medical reasons than gestational diseases (further described in the supplement information point 1.10), early-onset PE placental tissues (gestational age 24–33 weeks, *n* = 12), late third trimester controls (*n* = 6), and term PE placental tissues (gestational age 37–40 weeks, *n* = 6). The clinical information of these 27 patients is listed in Additional file 6 (Tab. S2) and Additional file 7 (Tab. S3), respectively. (4) hCV-MSCs for functional assays were isolated from 1st trimester (*n* = 5), term control (*n* = 4) and term PE placentas (*n* = 3). The clinical information of these 7 term control or term PE patients is summarized in Additional file 8 (Tab. S3). The samples of (2) and (3) as well as of (4) with exception of 1st trimester placentas were from the Department of Gynecology and Obstetrics, University Hospital Frankfurt. The collection of these samples was approved by the Ethics Committee of the Johann Wolfgang Goethe-University Hospital Frankfurt (reference number: 375/11). The 1st trimester placenta samples were provided by the Medical Practice for Gynecology and Obstetrics led by Dr. Nowak and the sample collection was approved by the Ethics Committee of the Johann Wolfgang Goethe-University Hospital Frankfurt (reference number: 19-455). Donor age was 28–41 (± 6.7) years, gestational age was 6–11 (± 2.1) weeks, and BMI was 23–31 (± 3.9). Informed written consent was obtained from all donors.

PE was diagnosed as an occurrence of hypertension after 20 weeks of gestation with a blood pressure ≥ 140/90 mmHg and proteinuria with ≥ 300 mg in 24 h. Patients with a concomitant HELLP syndrome were excluded from this study.

### Cell lines, organoid formation, spheroid formation, surface marker, cell viability, and cell cycle

Human villous cytotrophoblast stem cells (hTSCs^CT^) and human extravillous trophoblastic cell lines HTR-8/SVneo (referred to as HTR) and HIPEC-65 (referred to as HIPEC) were kindly provided by Dr. Okae and Prof. Arima [25], Prof. Graham [26], and Prof. Fournier [27], respectively. These cells were cultured as instructed [25–28].

Organoid formation, spheroid formation, surface marker characterization, cell viability, and cell cycle are detailed in supplementary information (Additional file 9) and as reported [17, 29–31].

### Isolation of hCV-MSCs

The isolation of hCV-MSCs was performed as reported [32] with modifications, described in supplementary information (Additional file 9).

### Isolation of human umbilical vein endothelial cells (HUVECs)

The isolation of HUVECs was performed as reported [33] with modifications, described in supplementary information (Additional file 9). The umbilical vein sample collection was approved by the Ethics Committee of the Johann Wolfgang Goethe-University Hospital Frankfurt (reference number: 19-201).

### Osteogenic, chondrogenic, and adipogenic differentiation of hCV-MSCs

hCV-MSC differentiation was performed with modifications, as reported [34, 35] and detailed in supplementary information (Additional file 9).

### Immunofluorescence staining of placental tissues and hCV-MSCs

For immunofluorescence staining, formalin-fixed, paraffin-embedded placental tissue sections were deparaffinized and stained as detailed in supplementary information (Additional file 9).

### Indirect immunofluorescence staining, imaging, and signal intensity measurement

The fluorescence staining and quantification were performed as reported [34, 36] and are explained in supplementary information (Additional file 9).

### Activation of the Hh pathway, zymography, and ELISA

The activation of the Hh pathway, zymography and ELISA were carried out as reported [34, 37, 38] and described in supplementary information (Additional file 9).

### RNA extraction and real-time PCR

Total RNAs were extracted from hCV-MSCs and EVT cell lines as described [39] and detailed in supplementary information (Additional file 9).

### Cellular network formation, cell motility, migration, and cell attraction

Cellular network formation, cell motility, migration, and cell attraction assays were performed as reported [17, 40, 41] and described in supplementary information (Additional file 9).

### Statistical analysis

Before statistical analysis, an outlier test was performed with all data sets. Student’s *t* test (two tailed and paired or homoscedastic) was used to evaluate the significance of difference between diverse groups for gene analysis, cell viability assay, cell cycle distribution, and ciliated cell population. The statistical evaluation of the single cell tracking assay, line-scan analysis, cellular network formation assay, and the measurement of the cilium length was performed by using an unpaired Mann-Whitney *U* test (two tailed). Difference is considered as statistically significant when *p* < 0.05.

## Results

### Placenta chorionic villous stroma cells are ciliated, the population of ciliated cells and their cilium lengths decrease during gestation

**Fig. 1:**
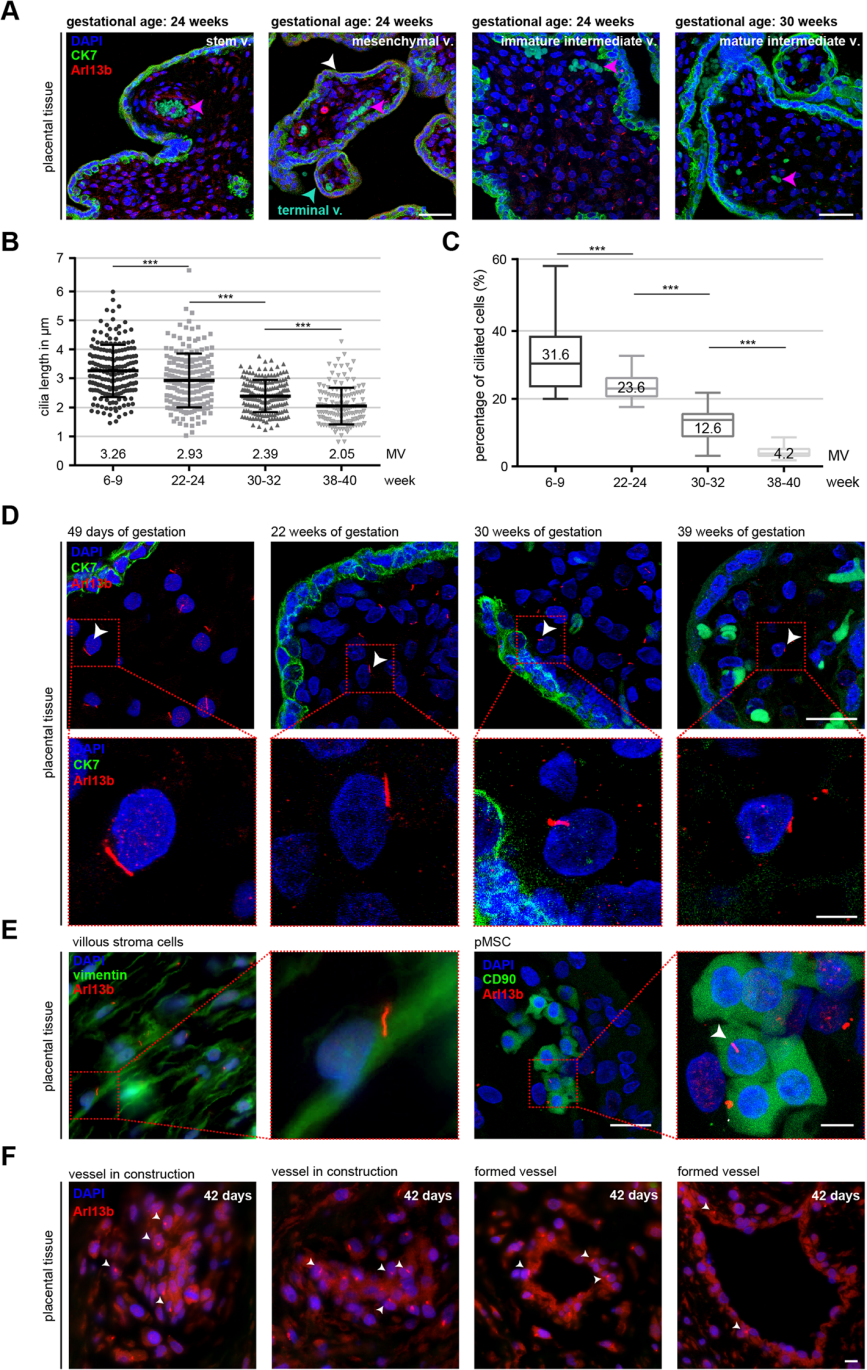
Villous stromal cells are ciliated and the ciliated population declines during gestation. **A** Placental tissues from 22 to 30 gestational weeks were stained for the primary cilium marker Arl13b (red), trophoblast marker cytokeratin 7 (green), and DNA (DAPI, blue). Representative ciliated stromal cells in stem villus, mesenchymal villus (white arrowhead), immature immediate villus, mature immediate villus, and terminal villus (green arrowhead) are shown. False positive staining of fetal red blood cells is indicated with a pink arrowhead. Scale: left two panels: 50 μm; right two panels: 25 μm. **B**–**D** Placental tissues from various gestational ages were stained for the Arl13b (red), cytokeratin 7 (green), and DNA (DAPI, blue). The cilium length of villous stromal cells and the percentage of ciliated cells were analyzed using the Arl13b staining. **B** The cilium length quantification is based on six different placental samples (*n* = 150–180 cilia for each group) and presented as scatter plot showing mean ± SEM. Unpaired Mann-Whitney *U* test was used. ∗∗∗*p* < 0.001. **C** The percentage of ciliated stromal cells in the villous cores was evaluated and is presented as median ± min/max whiskers in box plots (*n* = 600 cells, pooled from six experiments). Student’s *t* test was used. ∗∗∗*p* < 0.001. **D** Representatives of primary cilia are shown with indicated gestational age. Scale: 20 μm. Inset scale: 10 μm. **E** Placental tissue sections of 1st trimester placentas were also stained for mesenchymal cell marker vimentin (green), Arl13b (red) and DNA (blue) (left two panels) or with a mesenchymal stem/stromal cell marker CD90 (green), Arl13b (red), and DNA (blue) (right two panels). Representatives are shown. Scale: 20 μm. Inset scale: 10 μm. **F** Placental tissue sections from 1st trimester placentas, stained for Arl13b (red), cytokeratin 7 (green), and DNA (DAPI, blue), are shown as examples for the localization of ciliated stromal cells (arrowheads) near vessels in construction (**F**, 1st and 2nd panel) and formed vessels (vessel lumen < 5 μm) (**F**, 3rd and 4th panel). Scale: 20 μm

As we studied primary cilia in placental trophoblasts, we observed that stromal cells within the villi showed positive staining for primary cilium markers Arl13b and acetylated tubulin [17]. We wondered if villous stroma cells are ciliated among various villus types and whether ciliation occurs throughout the entire gestation period. To examine these issues, placental tissue sections at 22–30 gestational weeks, in which various villus types are easily found, were stained for Arl13b and cytokeratin 7, markers of trophoblastic cells, and evaluated microscopically. As depicted in Fig. 1A, most stromal cells within stem villi, mesenchymal villi, immature intermediate villi, mature intermediate villi, and terminal villi are ciliated, indicating the involvement of the primary cilium in the villus development. To address if primary cilia on those cells were dynamically regulated in terms of the length and percentage during pregnancy, placental tissue sections with various gestational ages were stained for Arl13b and cytokeratin 7 for analysis. Primary cilia were considerably present on the villous stromal cells throughout gestation (Fig. 1D). Further analysis revealed that the number of ciliated stromal cells within the villi declined significantly from the first-trimester to term placentas (31.6% vs. 4.2%) (Fig. 1C, D). In line with this observation, the length of the primary cilium decreased gradually as well (Fig. 1B, D). The mean length of the primary cilium on villous stromal cells was 3.26 μm in first-trimester placentas, which was progressively reduced to 2.05 μm in term placentas (Fig. 1B). Of these ciliated stromal cells, most were positively stained for vimentin (Fig. 1E, left two panels), a mesenchymal stromal cell marker, and some for cluster of differentiation 90 (CD90) (Fig. 1E, right two panels), a classical marker of MSCs. We observed a high number of ciliated villous cells clustered around vessels in construction (Fig. 1F, 1st and 2nd panels) and near the formed vessels (Fig. 1F, 3rd and 4th panels). Given that hCV-MSCs reside in perivascular niches in the placenta [42], it could be that some of them are hCV-MSCs. These findings indicate that most of the placental villous stromal cells are ciliated, which decline in number and length during gestation, implying its potential roles in placental development, especially in the early stages.

### Early-onset and term PE affects the primary cilium of hCV-MSCs

**Fig. 2:**
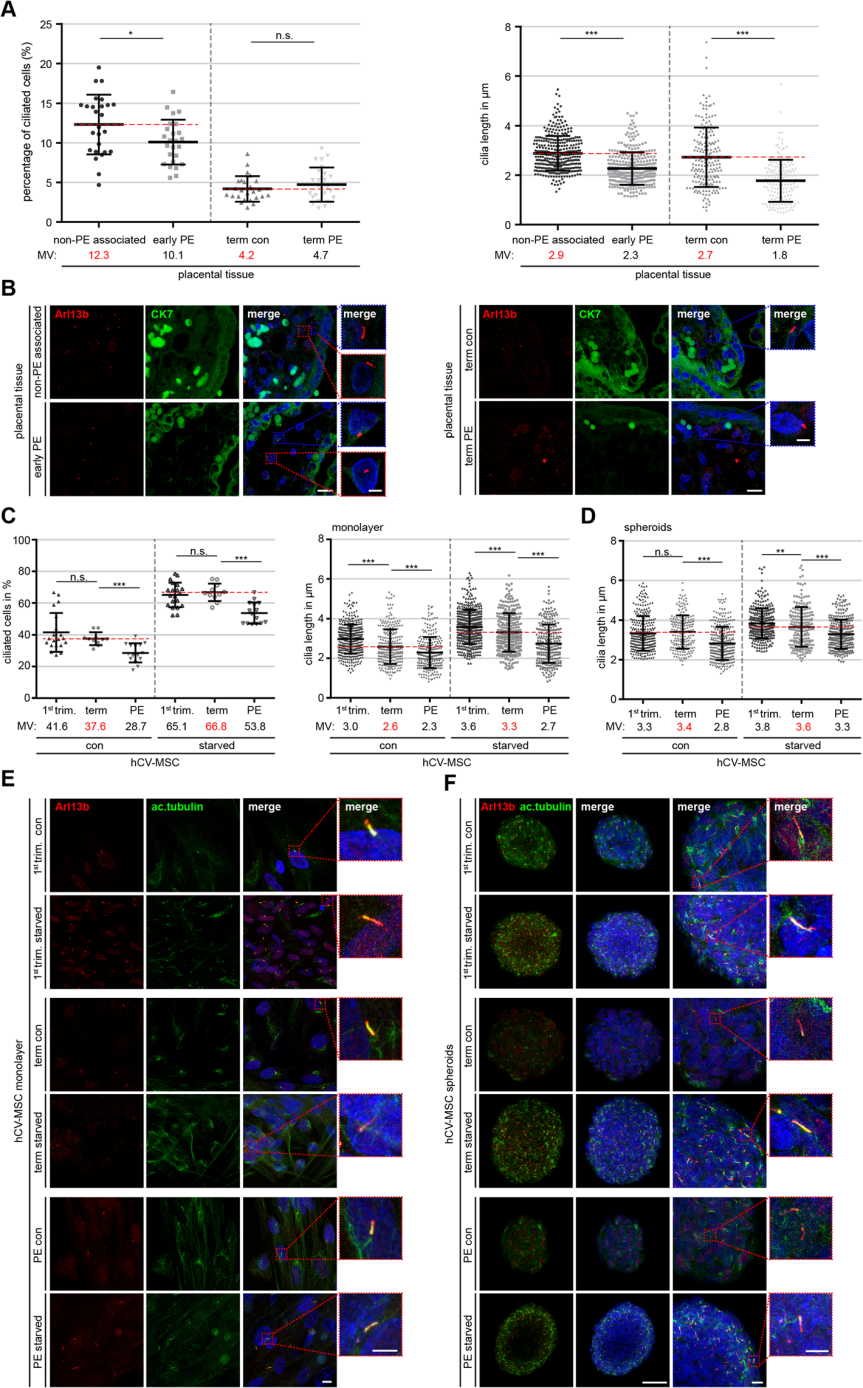
Primary cilia of hCV-MSCs from PE placentas are shortened. **A**, **B** Placental tissue sections from control and PE placentas were stained for Arl13b (red), acetylated α-tubulin (green), and DNA (DAPI, blue) or cytokeratin 7 (green) and Arl13b (red). The cilium length and the percentage of ciliated stroma cells within villi from early control (early con)/early-onset PE (early PE) placentas (gestational age < 37 weeks) and late control (late con)/term PE (term PE) placentas (gestational age ≥ 37 weeks) were evaluated by using the acetylated α-tubulin signal. **A** (left plot) The percentage of ciliated cells are presented as median ± min/max whiskers in box plots (*n* = 700 cells, pooled from seven different placentas for each group) and (**A**, right plot). The results of the cilium length are presented as scatter plot showing the mean ± SEM (*n* = 158–359 cilia, pooled from seven different placentas for each group). Unpaired Mann-Whitney *U* test was used in A and B. ∗*p* < 0.05, ∗∗∗*p* < 0.001. **B** Representative images of early control placental tissue and early-onset PE placental tissue (**B**, left panel), and term control placental tissue and term PE placental tissue (**B**, right panel) are shown. Scale: 20 μm. Inset scale: 10 μm. **C**–**F** Isolated hCV-MSCs from 1st trimester, term control and term PE placentas, non-treated or starved, were stained for Arl13b (red), acetylated α-tubulin (green), and DNA (DAPI, blue). The percentage of ciliated hCV-MSCs (**C**, left plot) and the cilium length (**C**, right plot) were evaluated. **C** The results of the ciliated cells in percentage (*n* = 15 fields of vision, pooled from three hCV-MSCs isolations for each group) and of the cilium length (*n* = 300 cilia, pooled from three hCV-MSCs isolations for each group) are presented as scatter plots showing mean ± SEM. **D** hCV-MSCs from 1st trimester, term control or term PE placentas formed the spheroids, which were non-treated or starved. The cilium length was evaluated and the results are presented as scatter plots showing mean ± SEM (*n* = 300 cilia, pooled from three hCV-MSCs isolations for each group). Unpaired Mann-Whitney *U* test was used in **C** and **D**. ∗∗*p* < 0.01, ∗∗∗*p* < 0.001. Representatives of stained hCV-MSCs, non-treated or starved in 2D and 3D culture, are shown (**E** and **F**). **E** Scale: 10 μm. Inset scale: 10 μm. **F** Scale: 50 μm. Inset scale: 10 μm. Smaller inset scale: 5 μm

We were also curious about what happened to villous ciliated cells in preeclamptic placentas since the primary cilium length is directly connected to its functionality, as shown especially in MSCs and other cell types [36, 43, 44]. In total, 32 placental tissue sections, comprising 12 early-onset PE placentas (< 34 GW), 8 early non-PE-associated placentas, 6 term PE placentas (≥ 37 GW), and 6 term control placentas (clinical information is listed in Tables S2 and S3), were stained for the cilium markers Arl13b and acetylated α-tubulin. Interestingly, the microscopic analysis of the cilium length on villous ciliated cells revealed significant reductions of 21.5% (2.9 vs. 2.3 μm) in early-onset PE placentas and 35.2% (2.7 vs. 1.8 μm) in term PE placentas compared to their matched control counterparts (Fig. 2A, left graph and B). Interestingly, the percentage of ciliated cells within the villi was significantly decreased by 18.1% in the early-onset PE samples compared to their control placentas, whereas there was hardly any difference observed between term PE placentas and their controls (4.7% vs. 4.2%, Fig. 2A, right graph). These results strongly suggest alterations to primary cilia on villous stromal cells in preeclamptic placentas.

To define the ciliated villous cells, primary hCV-MSCs were isolated from 1st trimester, term control, and term PE placentas. To characterize these cells, various cell surface markers were analyzed, showing a classical MSC expression profile with high expressions of CD90, CD73, and CD105 (Additional file 1, part B). Isolated hCV-MSCs were stained for cilium markers acetylated α-tubulin and Arl13b and microscopically analyzed. Interestingly, isolated primary hCV-MSCs showed a highly increased number of ciliated cells compared to cells in placental villi, which is in line with our previous observation for isolated primary hVCTBs [17]. hCV-MSCs derived from 1st trimester placentas showed a primary cilium on 41.6% of the cells, suggesting the significance of primary cilia in early placental development (Fig. 2C, left graph). Moreover, the ciliated hCV-MSC population was clearly reduced in term PE placentas, with 28.7%, compared to 37.6% in term control placentas (Fig. 2C, left graph). Additionally, hCV-MSCs from term PE placentas showed shorter cilia, with a mean value of 2.3 μm compared to 2.6 μm in control hCV-MSCs, in contrast to 3.0 μm in hCV-MSCs derived from 1st trimester placentas (Fig. 2C, right graph).

The assembly and disassembly of the primary cilium are coupled to the cell cycle [45]. To exclude the possibility that these cilium alterations are a result of changed cell cycle distribution and proliferation, FACS and cell titer assays were performed. The hCV-MSCs from term control placentas and those from term PE placentas showed no differences (Additional file 1, part A). Moreover, the primary cilium is dynamically assembled in the G_0_-phase [45]. To look precisely at the ciliary dynamics, hCV-MSCs were starved for 24 h for further analysis. Starvation increased the ciliated cell population of all three subgroups (by 65.1%, 66.8%, and 53.8% for the 1st trimester, term control, and term PE placentas, respectively), whereas PE hCV-MSCs significantly decreased in number (Fig. 2C, left graph and E). A similar observation was also made in terms of the cilium length. 1st trimester hCV-MSCs increased their primary cilium length from 3.0 μm to 3.6 μm (20.0%), term hCV-MSCs from 2.6 μm to 3.3 μm (26.9%), and PE hCV-MSCs from 2.3 μm to 2.7 μm (17.4%) (Fig. 2C, right graph). To mimic the physiological cellular environment in vivo, three-dimensional (3D) hCV-MSCs spheroids were formed for further investigations. Upon starvation, the mean cilium lengths increased to 3.8 μm, 3.6 μm, and 3.3 μm in hCV-MSCs from 1st trimester, term control, and term PE spheroids, respectively (Fig. 2D, F). These results indicate that the cilium assembly is impaired in PE hCV-MSCs. Starvation and spheroid culture could only partially restore the length of the primary cilium on PE hCV-MSCs but not to the extent of 1st trimester or term hCV-MSCs.

### Deficient Hedgehog (Hh) signaling in term PE hCV-MSCs

**Fig. 3:**
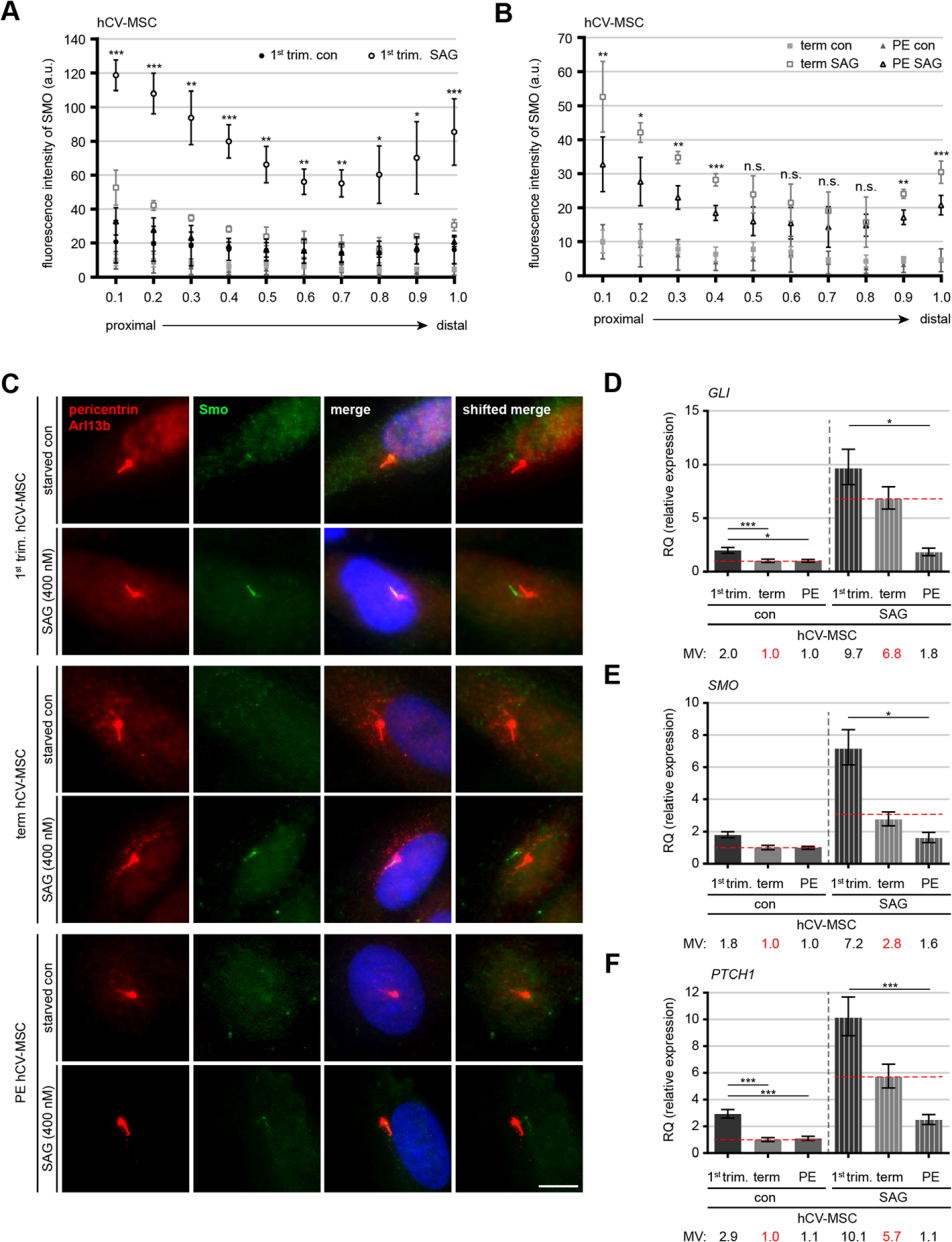
Deficient sonic hedgehog (Hh) pathway in term PE hCV-MSCs. **A**–**C** hCV-MSCs were starved for 24 h, stimulated with smoothened agonist (SAG, 400 nM) for 24 h and stained for the hedgehog marker Smo (green), cilia markers Arl13b (red), and pericentrin (red). Line-scan analyses of fluorescent Smo are shown as indicated for 1st trimester (**A**), term control and term PE hCV-MSCs (**B**). **A**, **B** Each point on the graph represents the mean fluorescence intensity and the results are presented as mean ± SEM (*n* = 30 cilia, pooled from three independent experiments). Unpaired Mann-Whitney *U* test was used. ∗*p* < 0.05, ∗∗*p* < 0.01, ∗∗∗*p* < 0.001. **C** Representatives of treated hCV-MSCs are shown. Scale: 10 μm. **D**–**F** Relative gene expression levels of *GLI1* (**D**), *SMO* (**E**), and *PTCH1* (**F**). Untreated term hCV-MSCs served as reference sample and GAPDH is used as endogenous control. Student’s *t* test was used. ∗*p* < 0.05, ∗∗*p* < 0.01, ∗∗∗*p* < 0.001

The Hh cascade requires the primary cilium for its function, as the regulators and receptors responsible for this signal transduction, such as Smoothened (Smo) and glioma-associated oncogene homolog 1 (Gli1), are solely located on the cilium membrane [46]. Because the Hh pathway modulates stem/progenitor cell differentiation [16, 47], we were interested in whether this pathway is still functional in hCV-MSCs from term PE placentas. To induce Hh activation, hCV-MSCs were stimulated for 24 h with Smoothened agonist (SAG), a Smo agonist that recruits Smo to the primary cilium [48]. After stimulation, cells were stained for Smo, pericentrin, and Arl13b for microscopic line-scanning analysis. Even without SAG stimulation, a weak Smo signal was already detectable on the proximal region of the cilium on hCV-MSCs from first-trimester placentas (Fig. 3A, C, 1st panel), whereas it was not found in the cilium of hCV-MSCs derived from term control placentas (Fig. 3B, C, 3rd panel). The stimulation with SAG induced an accumulation of Smo in the proximal and distal parts of the primary cilium (Fig. 3A–C). An in-depth line-scan analysis of the fluorescent signal revealed that hCV-MSCs from 1st trimester placentas demonstrated a highly increased Smo signal from the proximal region to the distal tip of the primary cilium, compared to hCV-MSCs from term control placentas (Fig. 3A vs. B, and C, 2nd vs. 4th panel), strengthening the importance of the Hh pathway in the early development of the placenta. Remarkably, primary cilia in term PE hCV-MSCs displayed significantly decreased Hh signaling activation compared to hCV-MSCs derived from the control placentas (Fig. 3B, C, 4th vs. 6th panel). Total RNAs were also isolated from untreated or SAG-treated hCV-MSCs and the expression of important Hh signaling genes was assessed via real-time quantitative PCR (qt-PCR). In line with the data from the fluorescent Smo signal quantification, the gene levels of *SMO* and its downstream targets, patched homolog 1 (*PTCH1*) and *GLI1*, were lower in hCV-MSCs derived from term PE placentas upon SAG stimulation compared to hCV-MSCs from term control placentas (Fig. 3D–F). In addition, the basic levels of the critical genes of the Hh pathway were higher in hCV-MSCs from 1st trimester placentas than in hCV-MSCs from term control and PE placentas (Fig. 3D–F), pointing to the significance of this pathway in the early-stage development of the placenta. In sum, these findings suggest an impairment of the Hh pathway in hCV-MSCs with shorter cilia from term PE placentas.

### Preeclampsia compromises the differentiation capacity of hCV-MSCs

**Fig. 4:**
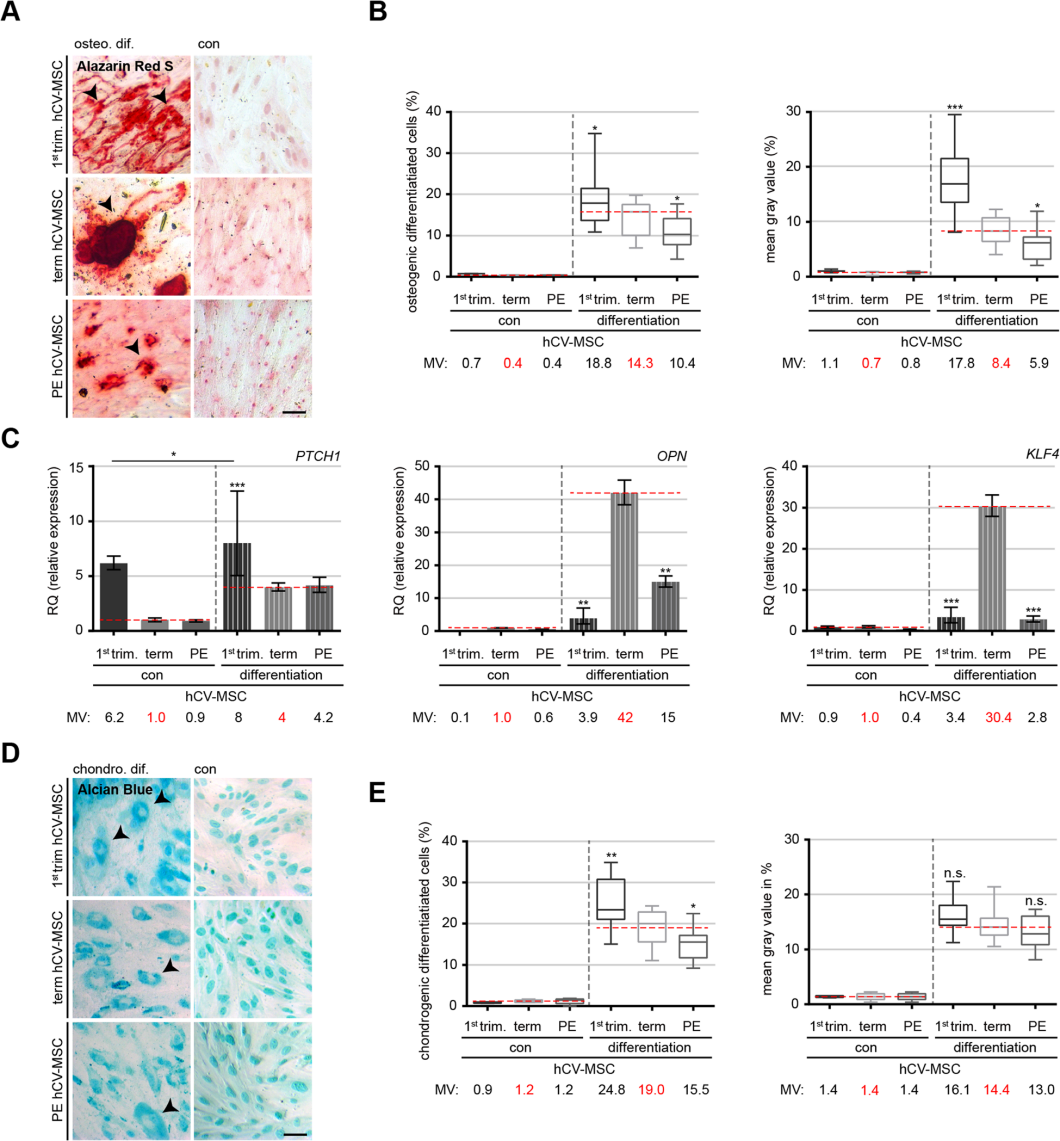
Preeclamptic hCV-MSCs differentiate poorly. **A**–**C** hCV-MSCs from 1st trimester, term control, and term PE placentas were subjected to osteogenic differentiation medium for 21 days. The percentage of differentiated osteogenic cells was evaluated by Alizarin Red S staining. **A** Representative images for Alizarin Red S staining are shown (arrowheads depict calcification). Scale: 20 μm. **B** The quantification of Alizarin Red S positive cells in percentage is shown as bar graph with mean ± SEM (**B**, 1st graph) as well as the mean gray value (**B**, 2nd graph) (*n* = 15, pooled from three independent experiments with three individual hCV-MSCs). **C** Expression levels of three osteogenic differentiation related genes *PTCH1* (left), *OPN* (middle), and *KLF4* (right) in control and hCV-MSCs upon differentiation. The results are from three independent experiments and presented as RQ with minimum and maximum range. **D**, **E** hCV-MSCs from 1st trimester, term control, and term PE placentas were subjected to chondrogenic differentiation medium for 21 days. The percentage of differentiated chondrogenic-like cells was evaluated by quantifying the Alcian Blue positive cells. **D** Representatives of chondrogenic-like cells stained by Alcian Blue are shown (blue: aggrecans). Scale: 50 μm. **E** Quantification of the mean gray value in percent (left) and the percentage of chondrogenic-like cells (right) are illustrated as bar graph showing mean ± SEM (*n* = 15, pooled from three independent experiments with three distinct hCV-MSCs for each group). Student’s *t* test was used in **C**. ∗*p* < 0.05, ∗∗*p* < 0.01, ∗∗∗*p* < 0.001. Unpaired Mann-Whitney *U* test was used in **B** and **E**. ∗*p* < 0.05, ∗∗*p* < 0.01, ∗∗∗*p* < 0.001

It is well documented that functional primary cilia are required for the differentiation of MSCs [34, 36, 49]. Because hCV-MSCs from PE placentas have shortened cilia and impaired Hh signal transduction, we were interested in their differentiation capacity. hCV-MSCs were subjected to osteogenic, chondrogenic, or adipogenic differentiation for 21 days. The differentiation was evaluated by corresponding cell type-specific markers. hCV-MSCs were stained for DNA and lipid vacuoles, which are characteristic of adipocytes, with Alizarin Red S, highlighting calcific deposits typical of osteogenic lineage, or with Alcian Blue to image acidic polysaccharides that are distinctive of chondrocytes. The positive stained cells and the staining intensities were evaluated. Notably, hCV-MSCs from 1st trimester placentas, which show longer cilia and a high percentage of ciliated cells, differentiated more efficiently into osteocytes and chondrocytes than term hCV-MSCs (Fig. 4). Patched homolog 1 (*PTCH1*), an important gene related to the osteogenic differentiation, was highly expressed in 1st trimester hCV-MSCs (Fig. 4C, left graph), whereas two other related genes, osteopontin (*OPN*) and Krüppel-like factor 4 (*KLF4*), did not seem to be required for their competent differentiation capability (Fig. 4C, middle and right graphs). Importantly, relative to term control hCV-MSCs, PE hCV-MSCs showed a decreased osteogenic differentiation capacity (Fig. 4A and B), along with reduced levels of *OPN* (Fig. 4C, middle graph) and *KLF4* (Fig. 4C, right graph). These data were further corroborated by a reduced percentage of Alizarin Red S-positive cells and a decreased gray mean value in term hCV-MSCs from term PE placentas compared to hCV-MSCs from control placentas (Fig. 4B). In further support, PE hCV-MSCs also displayed a reduced chondrocyte differentiation capacity (Fig. 4D, E). The percentage of differentiated chondrocytes was also significantly decreased in PE hCV-MSCs (Fig. 4E, left graph) as was the mean gray value of the Alcian Blue staining (Fig. 4E, right graph). It is well known that unlike adipose tissue-derived mesenchymal stem/stromal cells (ASCs) [50, 51], hCV-MSCs are reluctant to differentiate into adipocytes [52–54]. Indeed, upon differentiation, we observed only about 5% of adipocyte-like cells of all three hCV-MSCs subgroups, and no significant difference was observed among these subgroups (Additional file 1, part D and E). Moreover, the gene expression of *LEPTIN* was constantly upregulated in all three hCV-MSCs subgroups (Additional file 1, part F, left graph), and the gene *ADIPOQ*, coding for adiponectin, was higher, though not significantly, in term control hCV-MSCs compared to PE hCV-MSCs (Additional file 1, part F, right graph). Nevertheless, these results suggest that hCV-MSCs from term preeclamptic placentas differentiate poorly into all three lineages, which are likely associated with defective cilia, possibly contributing to impaired tissue repair or homeostasis in PE placentas.

### Preeclamptic hCV-MSCs decrease their own motility, their ability to stimulate mobility of trophoblastic cells and growth of human placental organoids

**Fig. 5:**
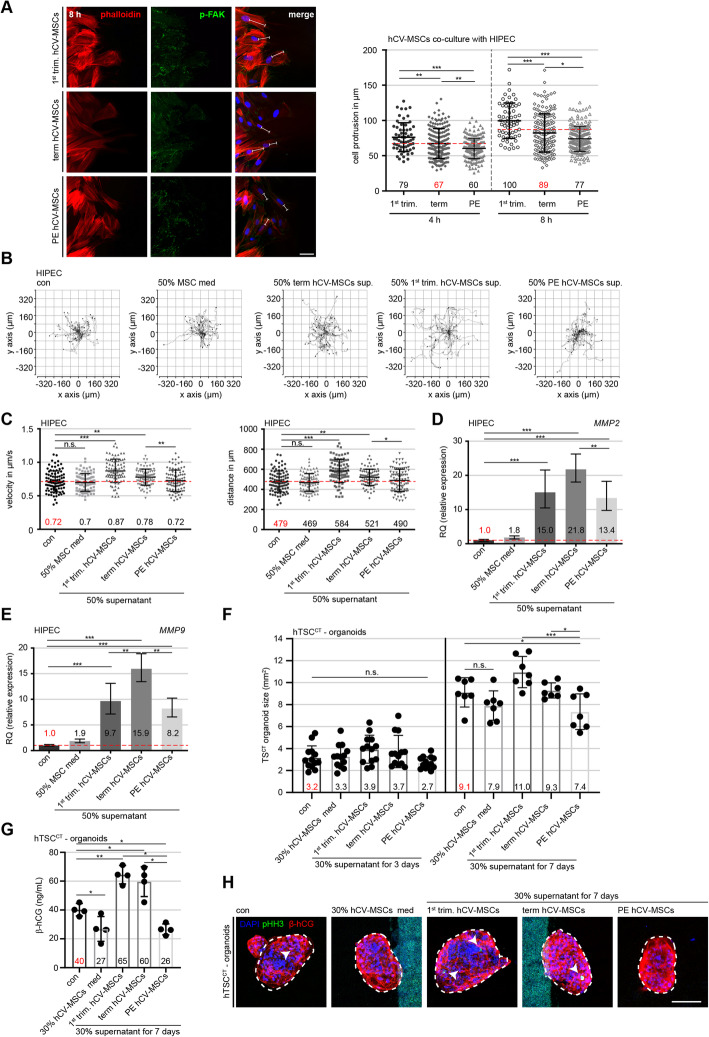
Impaired motility of PE hCV-MSCs and reduced stimulatory capacity for placental organoids and trophoblastic cells. **A** HIPEC cells and hCV-MSCs from 1st trimester, term control, and term PE placentas were seeded into each Ibidi chamber. After 8 h, the chambers were removed and the hCV-MSCs started to migrate toward HIPEC cells. After 4 and 8 h, bright-field images were taken for analysis. For fluorescence visualization, the cells were stained with phalloidin (actin filaments, red), p-FAK (focal adhesion marker, green), and DNA (DAPI, blue). Representatives of the hCV-MSCs at the migrating front after 8 h are shown (left). Scale: 50 μm. The length of the cellular protrusions of hCV-MSCs toward HTR cells was quantified and are presented as scatter plot (right) showing mean ± SEM (*n* = 180 protrusions, pooled from three independent experiments. **B**, **C** Single HTR cells were tracked after the treatment with indicated medium (control medium or medium containing 50% supernatant from MSCs of 1st trimester, term or term PE placentas) using time-lapse microscopy to analyze their cell motility. Representative trajectories are depicted for individual cells (**B**) (*n* = 30 cells in each group). The velocity (**C**, left graph) and accumulated distance (**C**, middle graph) are evaluated for each individual treatment and the results from three experiments are depicted as scatter plots showing mean ± SEM (*n* = 90 cells). Unpaired Mann-Whitney *U* test was used for (**A** and **C**). ∗*p* < 0.05, ∗∗*p* < 0.01, ∗∗∗*p* < 0.001. **D**, **E** HIPEC cells were incubated with indicated medium (control medium or medium containing 50% supernatant from MSCs of 1st trimester, term, or term PE placentas) for 7 days. Afterwards, total RNAs were extracted from treated HIPEC cells for analyzing gene levels of *MMP2* (**E**) and *MMP9* (**D**). The data are based on three independent experiments and presented as RQ with minimum and maximum range and statistically analyzed. RQ: relative quantification of the gene expression. **F**–**H** Placental organoids were generated for 72 h by using hTSC^CT^ cells and treated then for up to 7 days with 30% supernatants from 1st trimester, term or PE hCV-MSCs. The organoids were stained against β-hCG (red), pHH3 (green), and DNA (DAPI, blue) and microscopically evaluated. The results of the organoid area are presented as scatter graphs showing the mean ± SEM (*n* = 7-10 organoids, from three different hCV-MSC supernatants for each group) (**F**). Representative images of stained hTSC^CT^ organoids treated with different supernatants for 7 days are shown (white dotted lines indicate measured areas, arrowheads depict pHH3 positive cells). Scale: 200 μm. Supernatants of treated hTSC^CT^ organoids were also collected for the evaluation of β-hCG via ELISA (**G**). The results are from three experiments and presented as mean ± SEM in a scatter graph. Student’s t-test was used. ** *p* < 0.01, *** *p* < 0.001

The primary cilium has been shown to be associated with cell motility and the migration capacity of trophoblastic HTR cells and ASCs [17, 34, 55]. To examine this issue, single hCV-MSCs from 1st trimester, term control, and term PE placentas were tracked using time-lapse microscopy, which is a widely used assay for assessing cell motility by evaluating the accumulated distance and velocity of single-tracked cells [34, 40]. PE hCV-MSCs showed a reduced accumulated distance of 256 μm and 0.38 μm/s velocity, respectively, compared to term control hCV-MSCs, which had an accumulated distance of 348 μm and a velocity of 0.52 μm/s, whereas hCV-MSCs from 1st trimester placentas were the most motile cells, displaying an accumulated distance of 524 μm and velocity of 0.78 μm/s, an increase of 66.4% compared to term hCV-MSCs (Additional file 1, part C). Another important property of hCV-MSCs is their directed motility, also known as homing ability, toward other cell types, such as trophoblastic cells. This was analyzed by co-culturing hCV-MSCs with trophoblastic HIPEC cells separated by a defined gap between these two cell types. Co-cultured hCV-MSCs were stained for actin (phalloidin), phospho-focal adhesion kinase (pFAK), and DNA (Fig. 5A). The lengths of their cell protrusions toward HIPEC cells were measured after 4 h and 8 h of co-culture. In the first 4 h, only hCV-MSCs from 1st trimester placentas demonstrated a significant homing ability toward HIPEC cells, with a mean protrusion length of 79 μm, in contrast to mean lengths of 67 μm observed in the term hCV-MSCs and 60 μm in PE hCV-MSCs (Fig. 5A, scatter plot). After 8 h, PE hCV-MSCs displayed significantly shorter cell protrusions, with a mean length of 77 μm, compared to 89 μm in term hCV-MSCs, whereas hCV-MSCs from 1st trimester placentas were significantly attracted toward HIPEC cells (100 μm) (Fig. 5A, scatter plot). Similar results were also obtained with HTR cells, another trophoblastic cell line (Additional file 2, part A). These results suggest that hCV-MSCs from term PE placentas have impaired random as well as directed motility.

MSCs, including placental MSCs, are capable of producing a wide array of soluble growth factors and cytokines [56] to stimulate the migration and invasion of various cell types, including endothelial cells [57], breast cancer cells [50], and trophoblasts [58, 59]. To characterize this general capability of hCV-MSCs, single-cell time-lapse microscopy was used to assess their ability to influence the motility of trophoblastic HIPEC cells cultured with control medium, medium containing 50% supernatant from hCV-MSCs of 1st trimester, term control, or term PE placentas. Compared to control HIPEC cells, HIPEC cells treated with supernatants from PE hCV-MSCs increased their accumulated distance only by 2.3% (479 μm vs. 490 μm) and kept their velocity (0.72 μm/s vs. 0.71 μm/s), whereas supernatants from hCV-MSCs of term placentas increased their accumulated distance (479 μm vs. 521 μm) as well as their velocity (0.72 μm/s vs. 0.78 μm/s) by 8.8%. The strongest effect was observed with the supernatants of hCV-MSCs from 1st trimester placentas, which showed an increase of 21.9% in the accumulated distance (479 μm vs. 584 μm) and velocity (0.72 μm/s vs. 0.87 μm/s) (Fig. 5B, C). Comparable results were also obtained with HTR cells (Additional file 2, part B). To further investigate the effect of hCV-MSCs on the motility of trophoblastic cells, we conducted a migration assay with a direct cell-cell culture setup via an established wound-healing assay with defined cell chambers, where HTR or HIPEC cells were cultured and surrounded by 1st trimester hCV-MSCs, term hCV-MSCs, or PE hCV-MSCs (Additional file 3, part B). While hCV-MSCs from 1st trimester and term control placentas significantly improved the migration capacity of HTR and HIPEC cells, PE hCV-MSCs had no significant effect on this capacity of trophoblastic cells (Additional file 3, part B-F).

We investigated the possible mechanism by which trophoblastic cells meliorated their motility after co-culturing with normal hCV-MSCs. Matrix metallopeptidase 9 (MMP9) and MMP2 are regarded as key enzymes required to degrade the dense extracellular matrix inside the human placenta, particularly in the placental bed, for a proper invasion process of EVTs [60, 61]. The analysis of zymography showed that, compared to supernatants from term hCV-MSCs, HTR, or HIPEC cells co-cultured with supernatants from PE hCV-MSCs indicated a less active MMP2 and MMP9 (Additional file 2, part C and D), though without significance due to the variation induced by distinct hCV-MSCs from three individuals. Notably, trophoblastic cells treated with supernatants from 1st trimester hCV-MSCs showed highly active MMP2 and MMP9 (Additional file 2, part C and D).

In line with these results, the gene expressions of both *MMP2* and *MMP9* were increased in HIPEC or HTR cells cultured with supernatants of hCV-MSCs from 1st trimester or term placentas (Fig. 5D, E; Additional file 2, part E and F). By contrast, the expressions of these genes were significantly lower in trophoblastic cells treated with supernatants from PE hCV-MSCs (Fig. 5D, E; Additional file 2, part E and F). In addition, compared to term control hCV-MSCs, PE hCV-MSCs themselves showed a significantly lower gene expression of *MMP2* (Additional file 3, part G, left graph) and hardly detectable *MMP9* (Additional file 3, part G, right graph), which could explain the reduced migration of HTR cells after direct co-culture (Additional file 3, part C-F). Collectively, PE hCV-MSCs were significantly less capable of promoting the motility of trophoblasts, and had reduced expressions of *MMP2* and *MMP9*.

MSCs are well known for their ability to stimulate cell viability and proliferation and thus, they are regarded as promising tools for novel cell-based therapies [62]. To study if hCV-MSCs are able to promote the proliferation of trophoblasts, placental organoids were formed from hTSC^CT^ cells [30]. At 3 days, newly formed placental organoids were treated with 30% hCV-MSC supernatants for up to 7 days. The areas and diameters of these organoids were monitored by microscopy, and β-human chorionic gonadotropin (β-hCG) secretion, the indicator for trophoblast differentiation into hSTBs, was evaluated by ELISA assay. Compared to control organoids treated with 30% MSC medium for 7 days (7.9 mm^2^, Fig. 5F, H), hTSC^CT^ organoids treated with 30% supernatants from hCV-MSCs from 1st trimester and term placenta displayed increased organoid areas of 11.0 mm^2^ and 9.3 mm^2^, respectively (Fig. 5F, H). By contrast, organoids treated with 30% supernatants from PE hCV-MSCs showed areas of 7.4 mm^2^, even smaller than those of control organoids (7.9 mm^2^), though not significant (*p* = 0.059) (Fig. 5F, H). Comparable results were also obtained by measuring the organoid diameter after 7 days, which demonstrated significantly decreased diameters of hTSC^CT^ organoids treated with 30% supernatants from PE hCV-MSCs compared to organoids incubated with 30% supernatants from 1st trimester and term hCV-MSCs (Additional file 3, part A). Moreover, the secreted β-hCG by organoids treated with 1st trimester or term hCV-MSC supernatants was 65 ng/mL and 60 ng/mL compared to 40 ng/mL from the control organoids (Fig. 5G). Interestingly, organoids treated with supernatants from PE hCV-MSCs decreased their β-hCG secretion (26 ng/mL), as did the organoids treated with MSC medium (27 ng/ml) (Fig. 5G). To corroborate the stimulatory effect of hCV-MSC supernatants on placental organoids, we generated trophoblastic cell organoids by using JEG-3 cells as recently reported [31]. In line with the data from hTSC^CT^ organoids, the supernatants from 1st trimester and term hCV-MSCs increased significantly the organoid size compared to control organoids (Additional file 4, part A and B). By contrast, the supernatants of PE hCV-MSCs stimulated hardly cell proliferation in these organoids (Additional file 4, part A and B). Collectively, PE hCV-MSCs with shorter primary cilia lost their ability to promote proliferation and differentiation of hTSC^CT^ and JEG-3 cells in placental organoids, whereas normal hCV-MSCs did so with high competence, as reported for MSCs in various other cell types [62], highlighting their important role in regulating balanced tissue homeostasis.

### PE hCV-MSCs are less capable of facilitating the cellular network formation of HUVECs

**Fig. 6:**
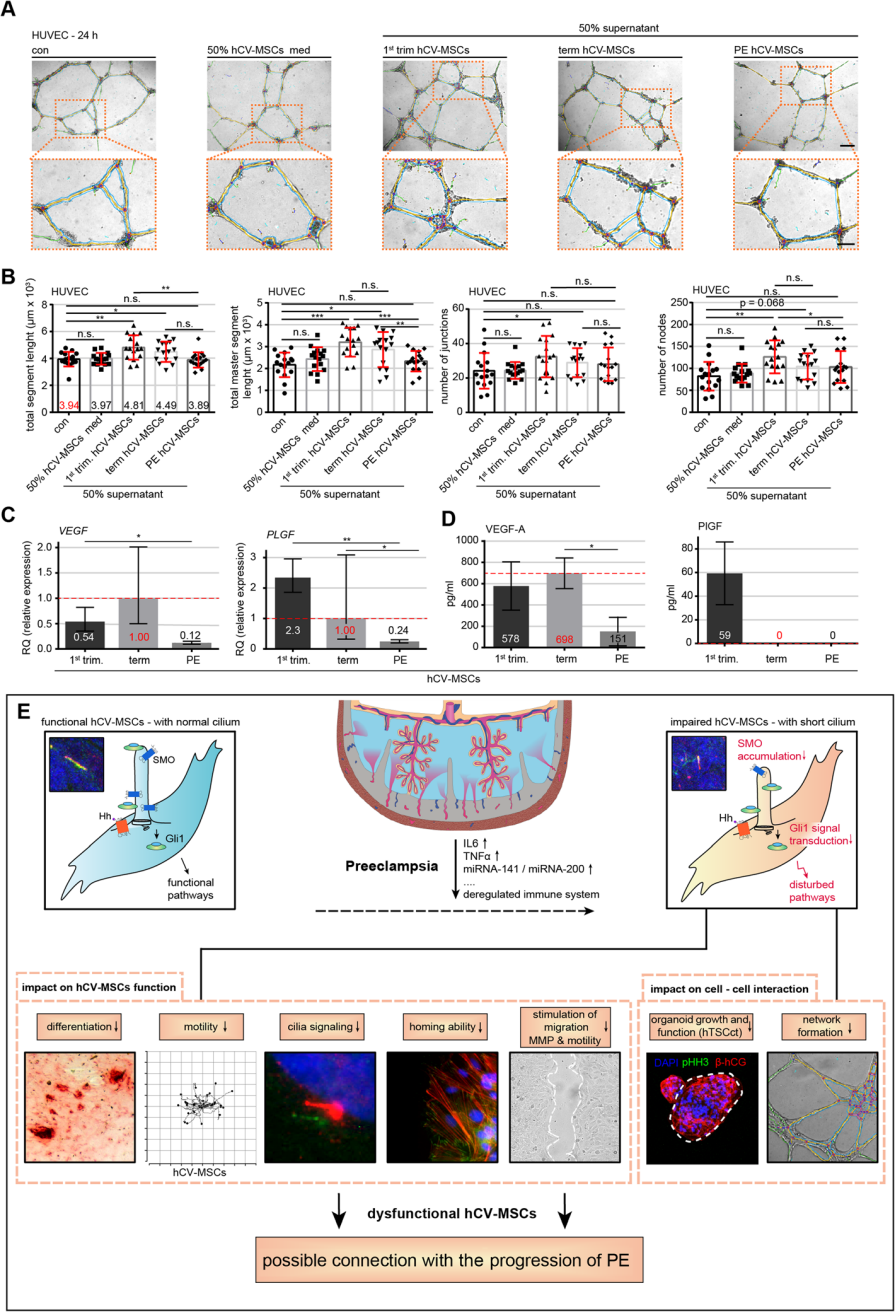
Preeclamptic hCV-MSCs are less capable of supporting cellular network formation of HUVECs. **A**, **B** The cellular network formation assay was performed with HUVECs cultured with different media as indicated (control medium, control medium containing 50% MSC normal medium, or containing 50% supernatants from hCV-MSCs of 1st trimester, term control, or term PE placentas). **A** Representatives of light microscopic images are shown (green: branches; cyan: twigs; yellow: master segments; red surrounded by blue: nodes surrounded by junctions; blue surrounded by red: master junctions). Scale: 200 μm. **B** Quantification of, the total segment length (**B**, 1st graph), total master segment length (**B**, 2nd graph), total number of junctions (**B**, 3rd graph), and total number of nodes (**B**, 4th graph) is shown. The results are based on three independent experiments (*n* = 15 pictures of each condition per group) and presented as bar graphs with mean ± SEM. **C** Total RNAs were extracted from hCV-MSCs as indicated for evaluating gene levels of *VEGF* (left) and *PLGF* (right). The results are from three experiments and presented as relative quantification of gene expression (RQ) with minimum and maximum range. **D** Secreted VEGF-A (left) and PlGF (right) were measured in supernatants from hCV-MSCs as indicated via ELISA. The results are based on three independent experiments and presented as bar graphs with mean ± SEM. Unpaired Mann-Whitney *U* test was used for (**B**), and Student *t* test was used in **C** and **D**. *p < 0.05, **p < 0.01, ***p < 0.001. **E** Schematic illustration of the proposed working model. PE associated factors like IL6 or TNFα shorten the primary cilium on hCV-MSCs, impairing their own motility, differentiation and signal transduction, and compromising their stimulatory capabilities in terms of cellular network formation of endothelial cells and growth/differentiation of placental organoids. The loss of functional hCV-MSCs with impaired cilia might be connected with progression of preeclampsia

We observed a high number of ciliated villous cells clustered around vessels (Fig. 1F) and hCV-MSCs have been reported to support placental vascular development [42]. To analyze if PE hCV-MSCs still possess this ability, we conducted a cellular network formation assay, a well-established assay to investigate the angiogenic potential of cells [63]. Isolated human primary umbilical vein endothelial cells (HUVECs) were seeded on Matrigel-coated plates and incubated for 12 h with different cell culture media; control medium, or medium containing 50% supernatants from 1st trimester, term, or PE hCV-MSCs. The ability to form a cellular network of treated cells was microscopically evaluated for the total segment length, total master segment length, number of junctions, and number of nodes in HUVEC cells. HUVECs were efficiently capable of branching into cellular network structures (Fig. 6A). Further analysis showed that supernatants from term and 1st trimester hCV-MSCs were able to promote HUVECs to form these structures (Fig. 6A, B), whereas the supernatant of PE hCV-MSCs showed fewer effects (Fig. 6A, B). These results were further corroborated with HTR cells (Additional file 4, part C and D). Moreover, gene analysis showed that, compared to term control hCV-MSCs, PE hCV-MSCs displayed decreased gene expressions of vascular endothelial growth factor (*VEGF*) and placental growth factor (*PLGF*) (Fig. 6C). In addition, these cells significantly reduced their secretion of VEGF-A (Fig. 6D, left graph). The significant effect on the cellular network formation of 1st trimester hCV-MSCs might be explained by their secretion of PlGF, which was not detected in term or PE hCV-MSC supernatants (Fig. 6D, right graph). In sum, the hCV-MSCs exerted a stimulatory effect on endothelial and EVT cells for their cellular network formation capacity. This effect was significantly reduced in hCV-MSCs from term PE placentas, likely due to a decreased secretion of factors such as VEGF-A and PlGF.

## Discussion

In the present work, we report that most villous stromal cells are ciliated within all villus types throughout gestation. It is of particular significance that primary cilia are abundantly present in stromal cells, including hCV-MSCs, in the villous core, as this population crucially supports placental development by influencing the morphogenesis of the branching architecture of the placenta and by driving placental vascular development [42]. Importantly, primary cilia on villous stromal cells are dynamically regulated in placental development, with a high percentage and the longest cilia length at the first weeks of gestation (6–9 weeks). The abundance of the primary cilium gradually declines till the end of pregnancy, strongly pointing to the notion that primary cilia on villous stromal cells may serve as processing centers for cell-cell signaling and cell-microenvironment communication [14].

Interestingly, the number and the length of primary cilia were significantly decreased in hCV-MSCs isolated from chorionic villi of term PE placentas. A possible explanation for the impaired ciliogenesis in preeclamptic hCV-MSCs could be an enhanced secretion of proinflammatory cytokines, including IL-6 and TNF-α, which are known to be upregulated in PE placental tissues and patient sera [17, 20, 64]. These cytokines could negatively affect the primary cilium of MSCs, as shown recently in trophoblasts isolated from preeclamptic patients, by increasing the expression of deciliation genes, including *KIF2A*, *PLK1*, and *AURKA*, or multiple deciliation microRNAs (miRNAs), such as miRNA-141 and miRNA-200 [17, 34, 43, 65]. Defective cilia, likely a consequence of PE, compromise the functionality of hCV-MSCs, as well as their interaction with other cells, which may further fuel the progression of PE.

As a consequence of an impaired cilium, the Hh signaling transduction of preeclamptic hCV-MSCs is significantly impaired, demonstrated by the reduced recruitment of Smo to the primary cilium and decreased levels of important related genes, such as *SMO*, *GLI1*, and *PTCH*. This defective signaling cascade is likely associated with the reduced differentiation capacity of PE hCV-MSCs since the primary cilium and its associated Hh pathway are important for osteo-, adipo-, and chondrogenic differentiation [36, 66]. Moreover, the Hh pathway is critical for faithful embryonic development, and its deregulation leads to severe diseases and tumorigenesis [46, 67]. The knockout of either sonic hedgehog or Gli2/Gli3 was shown to impair proper vasculogenesis and less branching, and led to the malformation of mouse placenta [68]. Additionally, the activation of the Hh pathway and its downstream target Gli2 stimulated the expression of 11β-Hydroxysteroid dehydrogenase type 2 required for a proper CTB fusion process [69]. The functionalities of EVTs were also associated with the Hh pathway. The Hh pathway triggered by the Forkhead box C2 was required for a correct epithelial-to-mesenchymal transition of EVTs to ensure a proper invasion into the maternal decidua [70]. These data suggest that the primary cilium, an exclusive mediator of the Hh pathway, is central for vital processes such as tissue homeostasis, vasculogenesis, invasion, differentiation, and fusion in human placenta. The impairment of these processes is correlated to defects in preeclamptic placentas, strongly indicating an association between the primary cilium and PE. More investigations are warranted to study hCV-MSCs from different gestational disorders to corroborate our observations and to unveil the related molecular connections.

MSCs are able to home and repair damaged tissues by secreting growth factors, including platelet-derived growth factor A/B (PDGFA/B), regenerative molecules, such as colony-stimulating factor 1 (CSF1), and anti-inflammatory cytokines, like IL-10 [71–73]. Our data show that, compared to hCV-MSCs from control placentas, hCV-MSCs from PE placentas have slower motility, indicating that these cells are less capable of participating in homing and repairing processes. Moreover, MSCs are known to enhance the motility of other cell types, including human alveolar and aortic endothelial cells, as well as malignant breast cancer cells, by the secretion of various cytokines, such as chemokine (C-X-C motif) ligand 1, monocyte chemoattractant protein 1 (MCP1), and TGF-β [74, 75]. Indeed, supernatants of 1st trimester and term hCV-MSCs significantly increased the motility of trophoblastic cells, whereas supernatants from PE hCV-MSCs were unable to do so. These findings are supported by the reduced gene expression and protein secretion of MMP-2 and MMP-9, both of which are closely associated with the invasion capacity of trophoblastic cells within the placental bed [76, 77]. Moreover, supernatants of term preeclamptic hCV-MSCs were less capable of promoting the cellular network formation of HUVECs and trophoblastic HTR cells. This is possibly associated with a decreased secretion of pro-angiogenic factors, such as VEGF-A, in preeclamptic hCV-MSCs. Finally, MSCs are well known to stimulate proliferation, enhance cell viability, and to promote the differentiation of various cell types in diverse tissues via paracrine signals, extracellular vesicles, or direct cell-cell contact [62, 78]. Our data support these observations by showing that 1st trimester and term hCV-MSCs were able to stimulate the growth and differentiation of hTSC^CT^ as well as JEG-3 organoids, whereas term PE hCV-MSCs failed to do so. PlGF or VEGF secreted by MSCs might be connected to the growth of these organoids, as has been described for trophoblasts [79–81].

## Conclusion

This work demonstrates the presence of the primary cilium within chorionic villi throughout the gestation of the human placenta and its functional significance. Importantly, primary cilia on hCV-MSCs from PE placentas are defective, which renders hCV-MSCs dysfunctional. Impaired hCV-MSCs with defective cilia might connect to compromised vasculogenesis and failed tissue homeostasis observed in PE placentas (Fig. 6E). Further studies are required to corroborate these findings. In addition, this work has several limitations, including the lack of a causative link between PE progression and defective cilia, small numbers of PE and control samples, term PE instead of early PE placenta-derived hCV-MSCs for functional analysis, and usage of the HTR cell line, which has been reported as a less valid cell model for human EVTs [82]. Finally, it is important to study the precise molecular mechanisms, how PE affects the formation and function of primary cilia on pMSCs, and how impaired pMSCs interact with and affect various placental cells in PE placentas.

## Supplementary Information

**Additional file 1: Figure S1.** hCV-MSCs from 1st trimester, term and PE placentas display comparable cell surface maker profiles, proliferation, and differentiation capacity. (A, left graph) Cell cycle distribution was analyzed using a FACSCaliburTM. The cell cycle phases of hCV-MSCs are presented in percentage and the results were derived from three independent experiments. (A, middle graph) Representative FL2-A histogram profiles of the cell cycle are shown. (A, right graph) hCV-MSCs were seeded in 96-well plates for 0, 24, 48, 72 and 96 h. Cell viability was measured via CellTiter-Blue® assay. The results are from three independent experiments and presented as mean ± SEM. *p < 0.05. (B) Flow cytometric analyses of positive cell surface markers CD90, CD73, CD105 and CD146, and negative markers CD14, CD31low/high, CD34, CD106, CD144 (B, left table). Representative staining of hCV-MSCs are shown for vimentin, actin and DNA (B, middle graph) or cell surface markers CD90 and CD73 (B, right graph). Scale: 20 μm. (C) Time-lapse microscopy was performed with hCV-MSCs for up to 12 h. Random motility of these cells was analyzed (*n* = 90 cells for each group). Representative trajectories of individual cells are shown (C, upper panels). Evaluated accumulated velocity (C, lower left plot) and distance (C, lower right plot) from three independent experiments are shown. Unpaired Mann–Whitney U-test, *** *p* < 0.001. (D-F) hCV-MSCs from 1st trimester, term and term PE placentas were subjected to adipogenic differentiation for 21 days. (D) Representative images for adipocytes are shown. Scale: 40 μm. Insert scale: 10 μm (arrowheads depict lipid vacuoles). (E) The percentage of differentiated adipocytes was evaluated by counting cells with lipid vacuoles. The quantification of cells displaying lipid vacuoles is shown as bar graph with mean ± SEM (*n* = 15, pooled from three independent experiments with three individual hCV-MSCs). (F) Expression levels of two adipogenic differentiation related genes LEPTIN (E, left graph), ADIPOQ (E, right graph) in control and hCV-MSCs upon differentiation. The results are from three independent experiments and presented as RQ with minimum and maximum range. Student’s t-test was used. ∗*p* < 0.05, ∗∗*p* < 0.01

**Additional file 2: Figure S2.** The motility and homing ability is impaired in PE hCV-MSCs as well as their capacity to stimulate motility and MMP2/9 activity in EVT cells. (A) HTR cells and hCV-MSCs from 1st trimester, term and term PE placentas were seeded into each Ibidi chamber. After 8 h, the chambers were removed and the hCV-MSCs started to migrate toward HTR cells. After 4 and 8 h bright-field images were taken for analysis. For fluorescence visualization, the cells were stained with phalloidin (actin filaments, red), p-FAK (focal adhesion marker, green) and DNA (DAPI, blue). (A, left panel) Representatives of the hCV-MSCs at the migrating front after 8 h are shown. Scale: 50 μm. (A, right graph) The length of the cellular protrusions of hCV-MSCs toward HTR cells was quantified, and was presented as scatter plot showing mean ± SEM (*n* = 180 protrusions, pooled from three independent experiments. (B) Single HTR cells were tracked after the treatment with indicated medium (control medium or medium containing 50% supernatant from hCV-MSCs of 1st trimester, term or term PE placentas) using time-lapse microscopy to analyze their cell motility. The velocity (C, left plot) and accumulated distance (C, right plot) were evaluated for each individual treatment. The results from three experiments are depicted as scatter plots showing mean ± SEM (*n* = 90 cells). Unpaired Mann-Whitney U test was used for (A and B). ∗p < 0.05, ∗∗*p* < 0.01, ∗∗∗*p* < 0.001. (C and D) HIPEC (C) and HTR (D) cells were incubated with indicated medium (control medium or medium containing 50% supernatant from hCV-MSCs of 1st trimester, term or term PE placentas) for 7 days. Afterwards, cells were starved-cultured for 24 h and the supernatants were collected for zymography assay to measure the activity of MMP2 and MMP9. (C and D, left panel) Representatives indicate the activity of MMP2 (lower band) and MMP9 (upper band). (C and D, middle and right graph) Quantification of MMP2 and MMP9 activity, normalized to the MSC control medium, is shown. The results are from three independent experiments and presented as bar graphs with mean ± SEM. (E) Total RNAs were also extracted from treated HTR cells for analyzing gene levels of MMP9 (E) and MMP2 (F). The data are based on three independent experiments and presented as RQ with minimum and maximum range and statistically analyzed. RQ: relative quantification of the gene expression. Student’s t-test was used. ** *p* < 0.01, *** *p* < 0.001

**Additional file 3: Figure S3.** hCV-MSCs from term PE placentas reduced their ability to support growth of placental organoids and migration of EVT cells. (A) Placental organoids were formed for 72 h by using hTSCCT cells and treated then for up to 7 days with 30% supernatant from 1st trimester, term control or term PE hCV-MSCs. The organoids were stained against β-hCG (red), pHH3 (green) and DNA (DAPI, blue), and their diameters were microscopically evaluated. The results are presented as scatter graphs showing the mean ± SEM (*n =* 7-10 organoids, from three different hCV-MSCs supernatants for each group). (B-F) Illustration of cell co-culture experiment. HIPEC/HTR cells (blue) were seeded into Ibidi chambers and surrounded by indicated hCV-MSCs from 1st trimester, term control and term PE placentas (red). After 8 h the chambers were removed, and the medium changed to HTR or HIPEC medium. Images were taken at indicated time points (0, 4, 8 h) to document the migration font. (B and D) Quantification of the open area between both migration fronts at various time points, for HTR (B) and HIPEC cells (D). The cell-free area at 0 h was assigned as 100%. The results from three independent experiments are presented as mean ± SEM. Unpaired Mann–Whitney U-test was used. *p < 0.05, **p < 0.01. (C and E) Representatives of the migration front are shown. White dashed line depicts the free area of the migration front. Scale: 200 μm. (G) Total RNAs were extracted from hCV-MSCs for analyzing gene levels of MMP2 (G, left graph) and MMP9 (G, right graph). The data are based on three independent experiments and presented as RQ with minimum and maximum range. RQ: relative quantification of the gene expression. Student’s t-test was used. *p < 0.05

**Additional file 4: Figure S4.** PE hCV-MSCs are less capable of supporting proliferation of JEG-3 organoids and network formation of HTR cells. (A and B) Placental organoids were generated for 96 h by using JEG-3 cells and treated then for up to 7 days with 30% supernatants from 1st trimester, term or PE hCV-MSCs. The organoids were stained against β-hCG (red), pHH3 (green) and DNA (DAPI, blue) and microscopically evaluated. The results of the organoid area are presented as scatter graphs showing the mean ± SEM (*n* = 5 organoids, from three different hCV-MSC supernatants for each group) (B). Representative images of stained JEG-3 organoids treated with different supernatants for 7 days are shown (white dotted lines indicate measured areas). Scale: 350 μm. Student’s t-test was used. ** *p* < 0.01. (C and D) Cellular network formation assay was performed with HTR cells cultured with different medium as indicated (control medium, control medium containing 50% MSC normal medium, or containing 50% supernatants from hCV-MSCs of 1st trimester, term or term PE placentas). (C) Representatives of light microscopic images are shown (green: branches; cyan: twigs; yellow: master segments; red surrounded by blue: nodes surrounded by junctions; blue surrounded by red: master junctions). Scale: 200 μm. (D) Quantification of total number of junctions (D, 1st graph), the total segment length (D, 2nd graph), total mesh area (D, 3rd graph) and total number of nodes (D, 4th graph) is shown. The results are based on three independent experiments (*n* = 15 pictures of each condition per group) and presented as bar graphs with mean ± SEM. Student’s t-test was used. *p < 0.05, ** *p* < 0.01, *** *p* < 0.001

**Additional file 5: Table S1.** Clinical information of patients with different gestational age, whose placental tissues were analyzed for cilium size and percentage. Mean value or value range ± standard deviation is shown

**Additional file 6: Table S2.** Clinical information of early-onset preeclampsia (PE) patients and matched controls, whose placental tissues were analyzed for cilium size and percentage. Mean value or value range ± standard deviation is shown

**Additional file 7: Table S3.** Clinical information of late-onset preeclampsia (PE) patients and matched controls, whose placental tissues were analyzed for cilium size and percentage. Mean value or value range ± standard deviation is shown

**Additional file 8:. Table S4.** Clinical information of term preeclampsia (PE) patients and matched controls, whose placental tissues were collected for the isolation of chorionic mesenchymal stem cells (hCV-MSCs). Mean value or value range ± standard deviation is shown

**Additional file 9.** Supplementary information.

## Data Availability

All data generated or analyzed during this study are included in this published article and its supplementary information files.

## Abbreviations

hCV-MSC: Human chorionic villi mesenchymal stromal cell
PE: Preeclampsia
hTSC: Human trophoblast stem cell
hVCTB: Human placental villous cytotrophoblast
hSTB: Human syncytiotrophoblast
hEVT: Extravillous trophoblast
hBD-MSC: Human basal decidua mesenchymal stromal cell
hCMSC: Human chorionic mesenchymal stromal cell
CIV-MSC: Chorionic cotyledons, intervillous space mesenchymal stromal cell
hCP-MSC: Human chorionic plate mesenchymal stromal cells
Hh: Sonic hedgehog
hTSC^CT^: Human villous cytotrophoblast stem cell
CD: Cluster of differentiation
3D: Three-dimensional
Smo: Smoothened
Gli1: Glioma-associated oncogene homolog 1
SAG: Smoothened agonist
qt-PCR: Real-time quantitative PCR
PTCH1: Patched homolog 1
OPN: Osteopontin
KLF4: Krüppel-like factor 4
ASC: Adipose tissue-derived mesenchymal stem/stromal cell
pFAK: Phospho-focal adhesion kinase
MMP: Matrix metallopeptidase
β-hCG: β-human chorionic gonadotropin
HUVEC: Human primary umbilical vein endothelial cell
VEGF: Vascular endothelial growth factor
PLGF: Placental growth factor
miRNA: microRNA
PDGFA/B: Platelet-derived growth factor A/B
CSF1: Colony-stimulating factor 1
MCP1: Monocyte chemoattractant protein 1
