# Supplementary figures and images for: Human placental mesenchymal stromal cells are ciliated and their ciliation is compromised in preeclampsia
Source: BMC Med. 2022 Jan 27;20:35. doi: 10.1186/s12916-021-02203-1 (PMC8793243; doi:10.1186/s12916-021-02203-1)

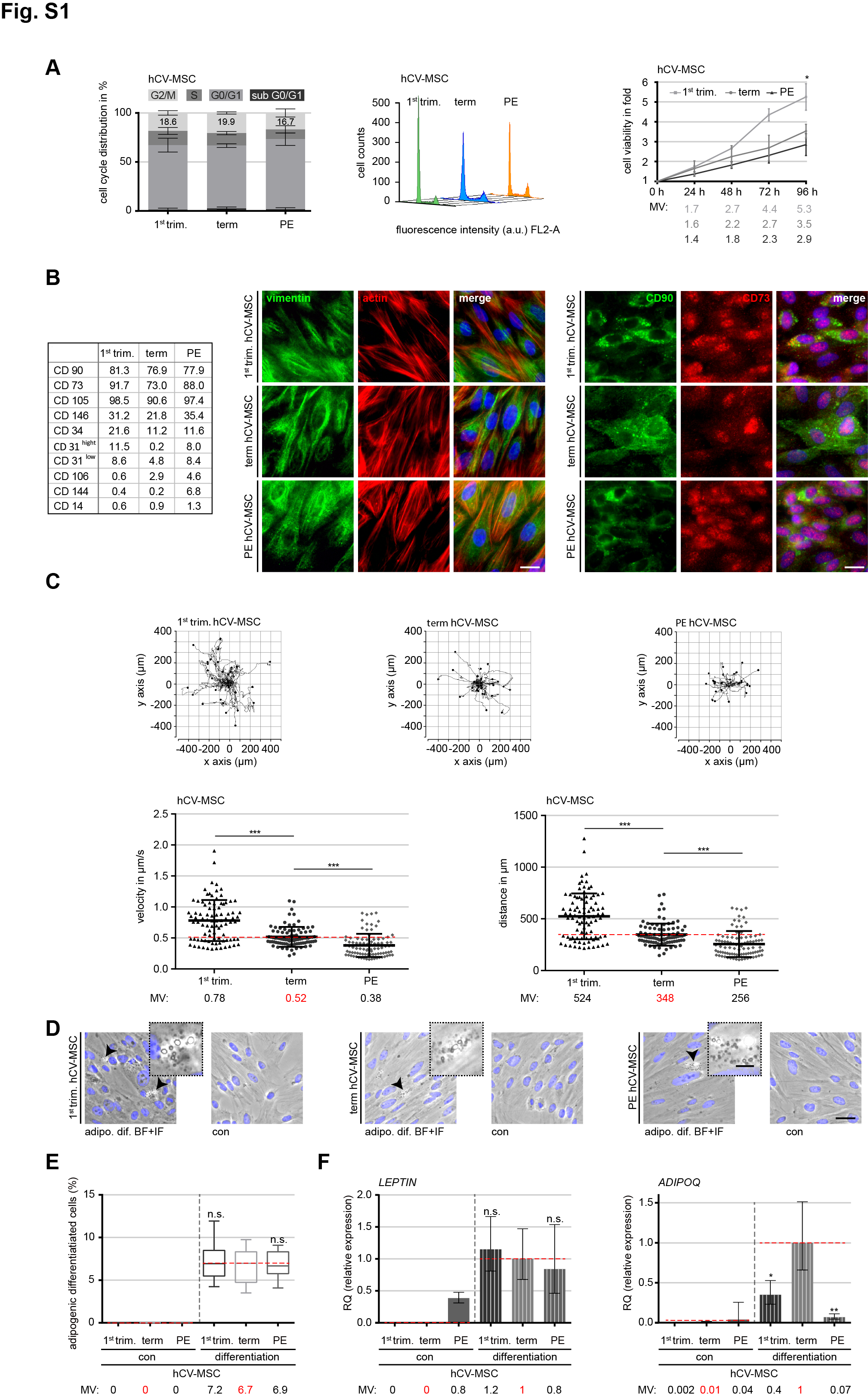

Supplement: Supplementary file 1 — Additional file 1: Figure S1. hCV-MSCs from 1st trimester, term and PE placentas display comparable cell surface maker profiles, proliferation, and differentiation capacity. (A, left graph) Cell cycle distribution was analyzed using a FACSCaliburTM. The cell cycle phases of hCV-MSCs are presented in percentage and the results were derived from three independent experiments. (A, middle graph) Representative FL2-A histogram profiles of the cell cycle are shown. (A, right graph) hCV-MSCs were seeded in 96-well plates for 0, 24, 48, 72 and 96 h. Cell viability was measured via CellTiter-Blue® assay. The results are from three independent experiments and presented as mean ± SEM. *p < 0.05. (B) Flow cytometric analyses of positive cell surface markers CD90, CD73, CD105 and CD146, and negative markers CD14, CD31low/high, CD34, CD106, CD144 (B, left table). Representative staining of hCV-MSCs are shown for vimentin, actin and DNA (B, middle graph) or cell surface markers CD90 and CD73 (B, right graph). Scale: 20 μm. (C) Time-lapse microscopy was performed with hCV-MSCs for up to 12 h. Random motility of these cells was analyzed (n = 90 cells for each group). Representative trajectories of individual cells are shown (C, upper panels). Evaluated accumulated velocity (C, lower left plot) and distance (C, lower right plot) from three independent experiments are shown. Unpaired Mann–Whitney U-test, *** p < 0.001. (D-F) hCV-MSCs from 1st trimester, term and term PE placentas were subjected to adipogenic differentiation for 21 days. (D) Representative images for adipocytes are shown. Scale: 40 μm. Insert scale: 10 μm (arrowheads depict lipid vacuoles). (E) The percentage of differentiated adipocytes was evaluated by counting cells with lipid vacuoles. The quantification of cells displaying lipid vacuoles is shown as bar graph with mean ± SEM (n = 15, pooled from three independent experiments with three individual hCV-MSCs). (F) Expression levels of two adipogenic differentiation r [file 12916_2021_2203_MOESM1_ESM.tif]

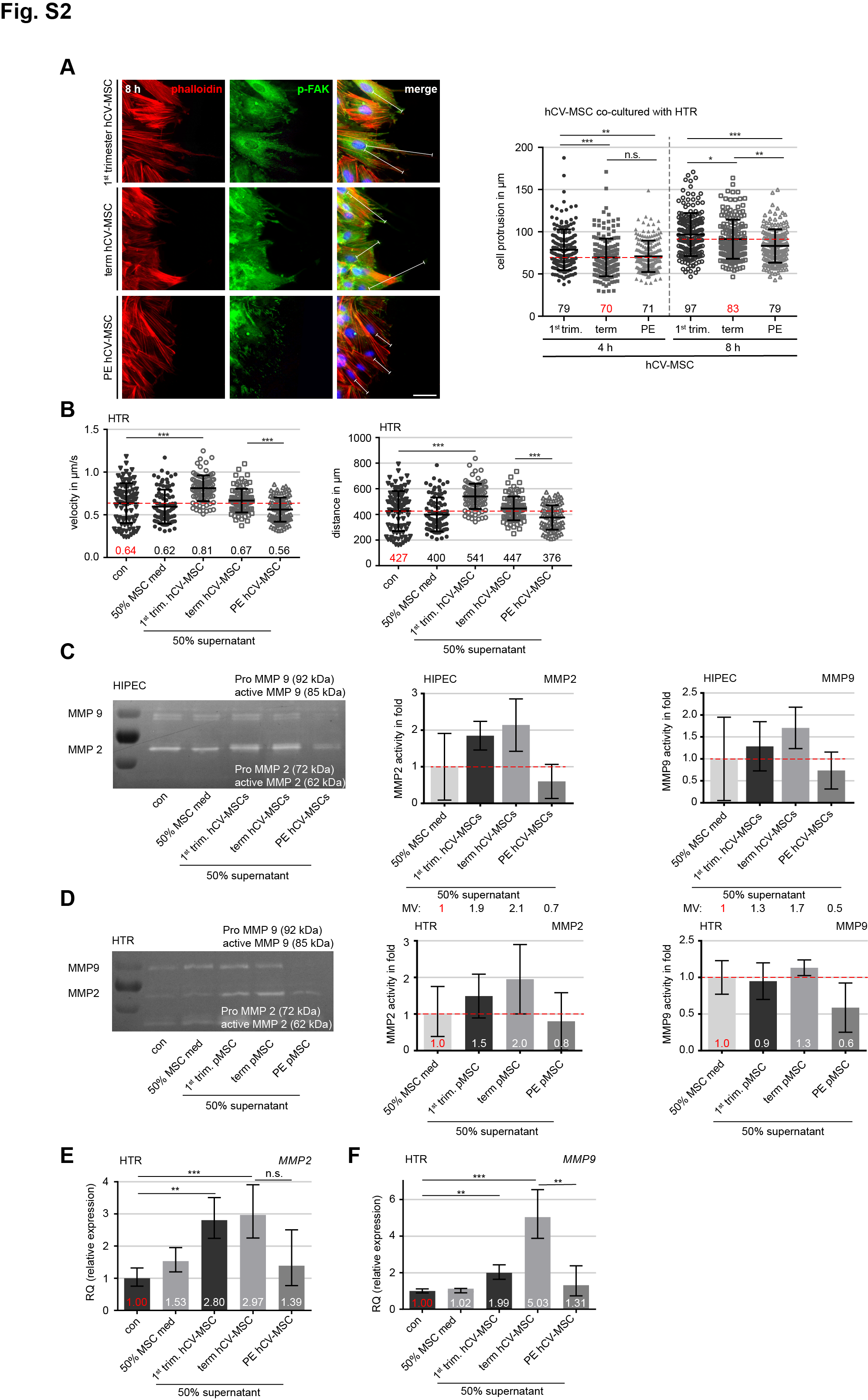

Supplement: Supplementary file 2 — Additional file 2: Figure S2. The motility and homing ability is impaired in PE hCV-MSCs as well as their capacity to stimulate motility and MMP2/9 activity in EVT cells. (A) HTR cells and hCV-MSCs from 1st trimester, term and term PE placentas were seeded into each Ibidi chamber. After 8 h, the chambers were removed and the hCV-MSCs started to migrate toward HTR cells. After 4 and 8 h bright-field images were taken for analysis. For fluorescence visualization, the cells were stained with phalloidin (actin filaments, red), p-FAK (focal adhesion marker, green) and DNA (DAPI, blue). (A, left panel) Representatives of the hCV-MSCs at the migrating front after 8 h are shown. Scale: 50 μm. (A, right graph) The length of the cellular protrusions of hCV-MSCs toward HTR cells was quantified, and was presented as scatter plot showing mean ± SEM (n = 180 protrusions, pooled from three independent experiments. (B) Single HTR cells were tracked after the treatment with indicated medium (control medium or medium containing 50% supernatant from hCV-MSCs of 1st trimester, term or term PE placentas) using time-lapse microscopy to analyze their cell motility. The velocity (C, left plot) and accumulated distance (C, right plot) were evaluated for each individual treatment. The results from three experiments are depicted as scatter plots showing mean ± SEM (n = 90 cells). Unpaired Mann-Whitney U test was used for (A and B). ∗p < 0.05, ∗∗p < 0.01, ∗∗∗p < 0.001. (C and D) HIPEC (C) and HTR (D) cells were incubated with indicated medium (control medium or medium containing 50% supernatant from hCV-MSCs of 1st trimester, term or term PE placentas) for 7 days. Afterwards, cells were starved-cultured for 24 h and the supernatants were collected for zymography assay to measure the activity of MMP2 and MMP9. (C and D, left panel) Representatives indicate the activity of MMP2 (lower band) and MMP9 (upper band). (C and D, middle and right graph) Quantification of MMP2 and MMP9 activity, normali [file 12916_2021_2203_MOESM2_ESM.tif]

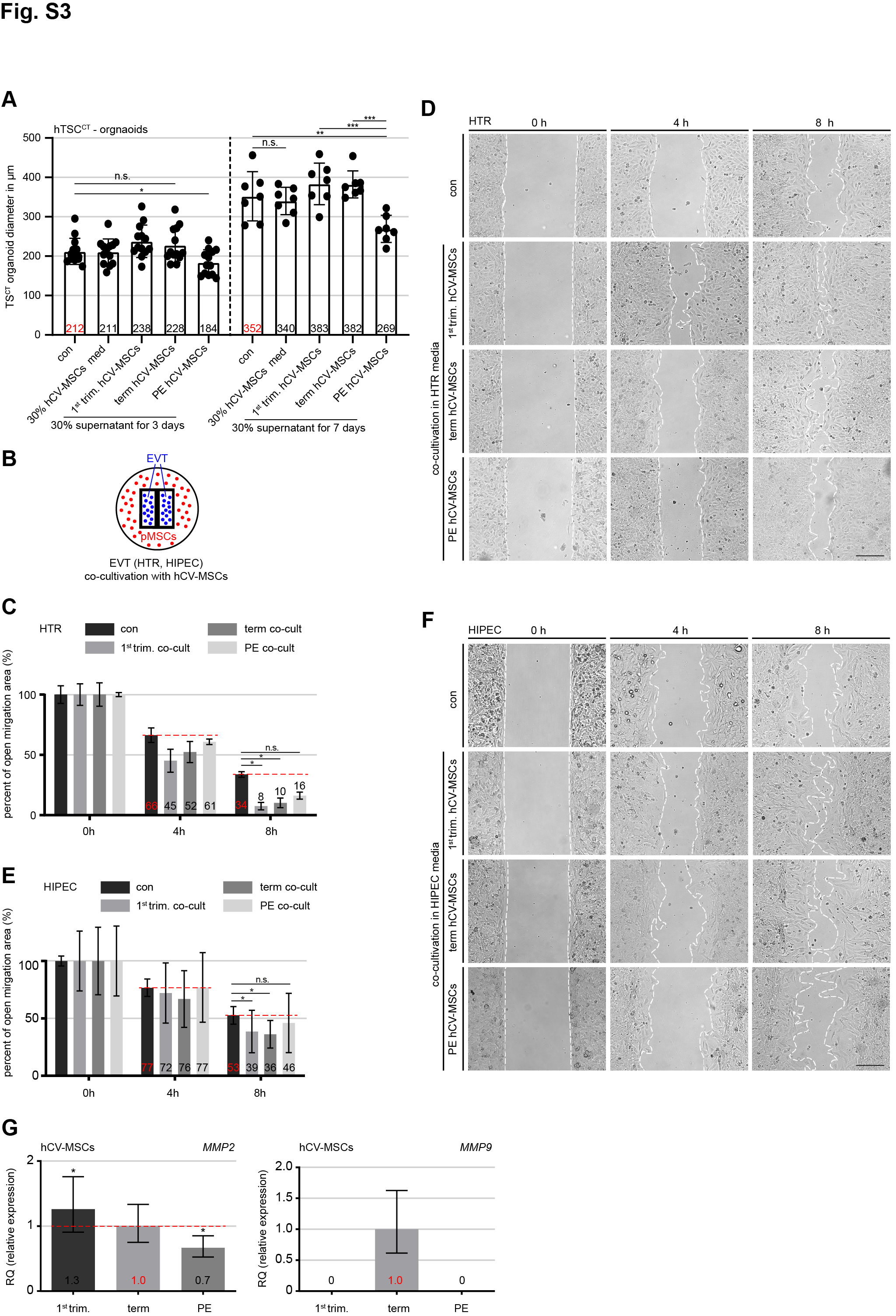

Supplement: Supplementary file 3 — Additional file 3: Figure S3. hCV-MSCs from term PE placentas reduced their ability to support growth of placental organoids and migration of EVT cells. (A) Placental organoids were formed for 72 h by using hTSCCT cells and treated then for up to 7 days with 30% supernatant from 1st trimester, term control or term PE hCV-MSCs. The organoids were stained against β-hCG (red), pHH3 (green) and DNA (DAPI, blue), and their diameters were microscopically evaluated. The results are presented as scatter graphs showing the mean ± SEM (n = 7-10 organoids, from three different hCV-MSCs supernatants for each group). (B-F) Illustration of cell co-culture experiment. HIPEC/HTR cells (blue) were seeded into Ibidi chambers and surrounded by indicated hCV-MSCs from 1st trimester, term control and term PE placentas (red). After 8 h the chambers were removed, and the medium changed to HTR or HIPEC medium. Images were taken at indicated time points (0, 4, 8 h) to document the migration font. (B and D) Quantification of the open area between both migration fronts at various time points, for HTR (B) and HIPEC cells (D). The cell-free area at 0 h was assigned as 100%. The results from three independent experiments are presented as mean ± SEM. Unpaired Mann–Whitney U-test was used. *p < 0.05, **p < 0.01. (C and E) Representatives of the migration front are shown. White dashed line depicts the free area of the migration front. Scale: 200 μm. (G) Total RNAs were extracted from hCV-MSCs for analyzing gene levels of MMP2 (G, left graph) and MMP9 (G, right graph). The data are based on three independent experiments and presented as RQ with minimum and maximum range. RQ: relative quantification of the gene expression. Student’s t-test was used. *p < 0.05 [file 12916_2021_2203_MOESM3_ESM.tif]

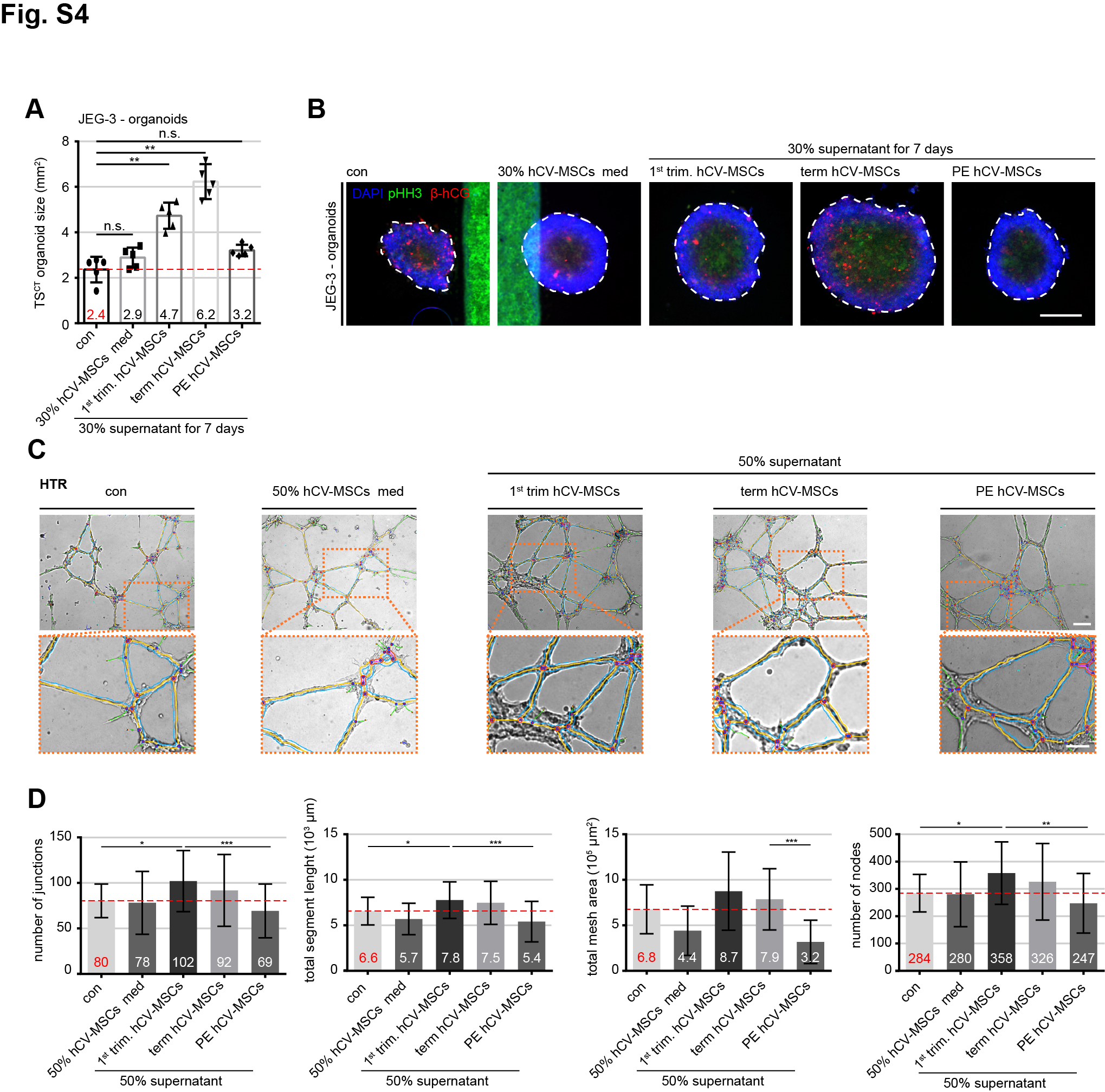

Supplement: Supplementary file 4 — Additional file 4: Figure S4. PE hCV-MSCs are less capable of supporting proliferation of JEG-3 organoids and network formation of HTR cells. (A and B) Placental organoids were generated for 96 h by using JEG-3 cells and treated then for up to 7 days with 30% supernatants from 1st trimester, term or PE hCV-MSCs. The organoids were stained against β-hCG (red), pHH3 (green) and DNA (DAPI, blue) and microscopically evaluated. The results of the organoid area are presented as scatter graphs showing the mean ± SEM (n = 5 organoids, from three different hCV-MSC supernatants for each group) (B). Representative images of stained JEG-3 organoids treated with different supernatants for 7 days are shown (white dotted lines indicate measured areas). Scale: 350 μm. Student’s t-test was used. ** p < 0.01. (C and D) Cellular network formation assay was performed with HTR cells cultured with different medium as indicated (control medium, control medium containing 50% MSC normal medium, or containing 50% supernatants from hCV-MSCs of 1st trimester, term or term PE placentas). (C) Representatives of light microscopic images are shown (green: branches; cyan: twigs; yellow: master segments; red surrounded by blue: nodes surrounded by junctions; blue surrounded by red: master junctions). Scale: 200 μm. (D) Quantification of total number of junctions (D, 1st graph), the total segment length (D, 2nd graph), total mesh area (D, 3rd graph) and total number of nodes (D, 4th graph) is shown. The results are based on three independent experiments (n = 15 pictures of each condition per group) and presented as bar graphs with mean ± SEM. Student’s t-test was used. *p < 0.05, ** p < 0.01, *** p < 0.001 [file 12916_2021_2203_MOESM4_ESM.tif]
