# Supplementary material for: Human placental mesenchymal stromal cells are ciliated and their ciliation is compromised in preeclampsia
Source: BMC Med. 2022 Jan 27;20:35. doi: 10.1186/s12916-021-02203-1 (PMC8793243; doi:10.1186/s12916-021-02203-1)
Supplement: Supplementary file 5 — Additional file 5: Table S1. Clinical information of patients with different gestational age, whose placental tissues were analyzed for cilium size and percentage. Mean value or value range ± standard deviation is shown [file 12916_2021_2203_MOESM5_ESM.docx]

**Supplementary table 1:** Clinical information of patients with different gestational age, whose placental tissues were analyzed for cilium size and percentage. Mean value or value range ± standard deviation is shown.

| **Group** | **n** | **Age (years)** | **Gestational age (weeks)** | **BMI** | **GP** | **Birth weight (g)** | **Systolic blood pressure** | **Diastolic blood pressure** | **Proteinuria** | **sFLT / PIGF** |
| --- | --- | --- | --- | --- | --- | --- | --- | --- | --- | --- |
| **22-24 weeks** | 4 | 31  ± 2.45 | 22-24  ± 1.15 | 29.7  ± 5.8 | < 3  ± 0 | 401  ± 73 | 111  ± 7.5 | 76  ± 3.5 | n.d. | n.d. |
| **30-32 weeks** | 6 | 33.5  ± 6.94 | 30-32  ± 0.84 | 23.96  ± 4.48 | < 3 - 34  ± 13.1 | 1545  ± 700 | 114.8  ± 6.7 | 70  ± 8.7 | n.d. | n.d. |
| **38-41 weeks** | 6 | 30.3  ± 4.18 | 38-41  ± 1.94 | 23.52  ± 8.22 | 19 – 85  ± 31.3 | 3442  ± 434 | 121.5  ± 10 | 74.75  ± 5.6 | n.d. | n.d. |

Abbreviation: n.d.: not determined, sFlt: Soluble Fms-like thyrosinkinase-1, PlGF: placental growth factor, GP: growth percentile.
