# Supplementary material for: Human placental mesenchymal stromal cells are ciliated and their ciliation is compromised in preeclampsia
Source: BMC Med. 2022 Jan 27;20:35. doi: 10.1186/s12916-021-02203-1 (PMC8793243; doi:10.1186/s12916-021-02203-1)
Supplement: Supplementary file 6 — Additional file 6: Table S2. Clinical information of early-onset preeclampsia (PE) patients and matched controls, whose placental tissues were analyzed for cilium size and percentage. Mean value or value range ± standard deviation is shown [file 12916_2021_2203_MOESM6_ESM.docx]

**Supplementary table 2:** Clinical information of early-onset preeclampsia (PE) patients and matched controls, whose placental tissues were analyzed for cilium size and percentage. Mean value or value range ± standard deviation is shown.

| **Group** | **n** | **Age (years)** | **Gestational age (weeks)** | **BMI** | **GP** | **Birth weight (g)** | **Systolic blood pressure** | **Diastolic blood pressure** | **Proteinuria** | **sFLT / PIGF** |
| --- | --- | --- | --- | --- | --- | --- | --- | --- | --- | --- |
| **Non-PE**  **associated** | 8 | 33.9  ± 3.5 | 26 - 34  ± 2.9 | 25.5  ± 4.0 | < 3 - 57  ± 21.6 | 1324  ± 766 | 127  ± 13 | 79  ± 9 | n.d. | n.d. |
| **Early-onset**  **PE** | 12 | 31.8  ± 5.9 | 25 - 32  ± 2.6 | 24.7  ± 4.9 | < 3 - 34  ± 9.6 | 1022  ± 370 | 156  ± 32 | 93  ± 16 | 3235  ± 4570 | 314  ± 107 |
| ***p*-Value** |  | 0.368 | 0.355 | 0.154 | 0.037 | 0.301 | 0.026 | 0.033 | n.d. | n.d. |

Abbreviation: n.d.: not determined, sFlt: Soluble Fms-like thyrosinkinase-1, PlGF: placental growth factor, PE: Preeclampsia, GP: growth percentile.
