# Supplementary material for: Human placental mesenchymal stromal cells are ciliated and their ciliation is compromised in preeclampsia
Source: BMC Med. 2022 Jan 27;20:35. doi: 10.1186/s12916-021-02203-1 (PMC8793243; doi:10.1186/s12916-021-02203-1)
Supplement: Supplementary file 7 — Additional file 7: Table S3. Clinical information of late-onset preeclampsia (PE) patients and matched controls, whose placental tissues were analyzed for cilium size and percentage. Mean value or value range ± standard deviation is shown [file 12916_2021_2203_MOESM7_ESM.docx]

**Supplementary table 3:** Clinical information of late-onset preeclampsia (PE) patients and matched controls, whose placental tissues were analyzed for cilium size and percentage. Mean value or value range ± standard deviation is shown.

| **Group** | **n** | **Age (years)** | **Gestational age (weeks)** | **BMI** | **GP** | **Birth weight (g)** | **Systolic blood pressure** | **Diastolic blood pressure** | **Proteinuria** | **sFLT / PIGF** |
| --- | --- | --- | --- | --- | --- | --- | --- | --- | --- | --- |
| **Control** | 6 | 38.4  ± 1.52 | 35 - 40  ± 1.94 | 23.52  ± 8.22 | 6 - 30  ± 20.5 | 2692  ± 419 | 121.5  ± 10 | 74.75  ± 5.6 | n.d. | n.d. |
| **Term**  **PE** | 6 | 30.17  ± 3.18 | 37 - 40  ± 1.63 | 23.23  ± 1.48 | < 3 - 24  ±  8.3 | 2288  ± 406 | 166.5  ± 26 | 104.7  ± 22 | 1864  ± 1914 | 273  ± 139 |
| ***p*-Value** |  | 0.47 | 0.44 | 0.47 | 0.14 | 0.066 | 0.006 | 0.017 | n.d. | n.d. |

Abbreviation: n.d.: not determined, sFlt: Soluble Fms-like thyrosinkinase-1, PlGF: placental growth factor, PE: Preeclampsia, GP: growth percentile.
