# Supplementary material for: Human placental mesenchymal stromal cells are ciliated and their ciliation is compromised in preeclampsia
Source: BMC Med. 2022 Jan 27;20:35. doi: 10.1186/s12916-021-02203-1 (PMC8793243; doi:10.1186/s12916-021-02203-1)
Supplement: Supplementary file 8 — Additional file 8:. Table S4. Clinical information of term preeclampsia (PE) patients and matched controls, whose placental tissues were collected for the isolation of chorionic mesenchymal stem cells (hCV-MSCs). Mean value or value range ± standard deviation is shown [file 12916_2021_2203_MOESM8_ESM.docx]

**Supplementary table 4:** Clinical information of term preeclampsia (PE) patients and matched controls, whose placental tissues were collected for the isolation of chorionic mesenchymal stem cells (hCV-MSCs). Mean value or value range ± standard deviation is shown.

| **Group** | **n** | **Age (years)** | **Gestational age (weeks)** | **BMI** | **GP** | **Birth weight (g)** | **Systolic blood pressure** | **Diastolic blood pressure** | **Proteinuria** | **sFLT / PIGF** |
| --- | --- | --- | --- | --- | --- | --- | --- | --- | --- | --- |
| **Control** | 4 | 30.5  ± 3.9 | 40 - 42  ± 0.96 | 21.73± 2.10 | 13 – 86 ± 35.9 | 3273  ± 539 | 123.5  ± 3.42 | 76.5  ± 10.28 | n.d. | n.d. |
| **Term**  **PE** | 3 | 32.67  ± 3.06 | 37 - 40  ± 1.53 | 25.8 ± 5.09 | 3 – 64 ± 29.7 | 2573 ± 595 | 171  ± 6.56 | 108.3  ± 6.66 | 4420  ± 3465 | 310.33 ± 118.89 |
| ***p*-Value** |  | 0.355 | 0.009 | 0.135 | 0.151 | 0.039 | 0.0004 | 0.0028 | n.d. | n.d. |

Abbreviation: n.d.: not determined, sFlt: Soluble Fms-like thyrosinkinase-1, PlGF: placental growth factor, PE: Preeclampsia, GP: growth percentile.
