# Supplementary material for: Human placental mesenchymal stromal cells are ciliated and their ciliation is compromised in preeclampsia
Source: BMC Med. 2022 Jan 27;20:35. doi: 10.1186/s12916-021-02203-1 (PMC8793243; doi:10.1186/s12916-021-02203-1)
Supplement: Supplementary file 9 — Additional file 9. Supplementary information. [file 12916_2021_2203_MOESM9_ESM.docx]

Online Data Supplement

**Human placental mesenchymal stromal cells are ciliated and their ciliation is impaired in preeclampsia**

Sophia Indira Romberg^1^, Nina-Naomi Kreis^1^, Alexandra Friemel^1^, Susanne Roth^1^, Alice Steglich Souto^1^, Samira Catharina Hoock^1^, Kyra Fischer^1^, Thorsten Nowak^2^,

Christine Solbach^1^, Frank Louwen^1^, Andreas Ritter^1, #^ and Juping Yuan^1, #^

^1 Division of Obstetrics and Prenatal Medicine, Department of Gynecology and Obstetrics, University Hospital Frankfurt, J. W. Goethe- University, Theodor-Stern-Kai 7, D-60590 Frankfurt, Germany^

^2 Medical practice for Gynecology, Mainzer Landstraße 265, D-60326 Frankfurt, Germany^

^# contributed equally as last author.^

^* Correspondence: andreas.ritter@kgu.de and yuan@em.uni-frankfurt.de^

Running title: Primary cilia in placental mesenchymal stem/stromal cells

**Corresponding Authors:**

Dr. Andreas Ritter: Email: andreas.ritter@kgu.de; Tel.: +49 69 6301 83297

Dr. Juping Yuan: Email: yuan@em.uni-frankfurt.de; Tel.: +49 69 6301 5819

Division of Obstetrics and Prenatal Medicine, Department of Gynecology and Obstetrics, University Hospital Frankfurt, J. W. Goethe-University, Theodor-Stern-Kai 7, D-60590 Frankfurt, Germany

**Co-Authors:**

Sophia Indira Romberg: sophia.i.romberg@med.uni-giessen.de; Nina-Naomi Kreis: nina-naomi.kreis@kgu.de; Alexandra Friemel: alexandra.friemel@kgu.de; Susanne Roth: susanne.roth@kgu.de; Alice Steglich Souto: alice.steglichsouto@kgu.de; Samira Catharina Hoock: samiracatharina.hoock@kgu.de; Kyra Fischer: kyra.fischer@kgu.de; Thorsten Nowak: info@dr-nowak.net; Christine Solbach: christine.solbach@kgu.de; Frank Louwen: louwen@em.uni-frankfurt.de

We declare no conflict of interest, both financial and personal.

**1. Materials and Methods**

**1.1 Organoid formation, spheroid formation, surface marker, cell viability and cell cycle**

Organoids were formed by using hTSC^CT^ (4 x 10^3^ cells) or JEG-3 cells (5 x 10^3^ cells) as detailed by Castel et al. 2021 and Dietrich et al. 2021. hTSC^CT^ cells were seeded on low attachment plates and treated for 3 days with trophoblast organoid medium (TOM): [DMEM/F12, 1x N2 supplement, 1x B27 supplement minus vitamin A, 1.25 mM N-Acetyl-L-cysteine, 1% GlutaMAX (Gibco), 0.5% Penicillin-Streptomycin (TOM basal medium), supplemented with 500 nM A83-01, 1.5 mM CHIR99021, 80 ng/ml human R-spondin1, 50 ng/ml hEGF, 100 ng/ml hFGF2, 50 ng/ml hHGF, 2 mM Y-27632]. The generation of JEG-3 organoids was performed similar with the following medium: [advanced DMEM (Gibco), 1x B27 supplement minus vitamin A, 10 mM HEPES, 2 mM glutamine (Gibco), 1 x Insulin- Transferrin-Selenium-Ethanolamine (ITS-X), 1% Penicillin-Streptomycin, 1 µM A83-01 (Tocris), 3 µM CHIR99021 (Tocris) and 100 ng/ml hEGF (R&D Systems]. After three days (hTSC^CT^) or four days (JEG-3) an initial 3D structure was formed, the medium was then supplemented with 30% of hCV-MSCs supernatants from 1^st^ trimester, term control or term PE placenta, or 30% hCV-MSC normal medium for 7 days. Organoids were stained for β-hCG, pHH3 and DNA, and the areas and diameters of placental organoids were monitored via microscopy.

Spheroids were formed by using a hanging drop system (2 x 10^3^ cells in 25 µl droplets) in hCV-MSC medium. After 72 h to 96 h of formation, the spheroids were transferred into a 4-chamber slide and the medium was changed for starvation (DMEM without FCS (Gibco, Carlsbad, USA, #11966025)). After another 24 h, the cells were fixed and stained.

For the treatment with conditioned medium, HTR/HIPEC cells (3 x 10^4^ cells) were seeded into six well plates with control medium, medium consisting of 50% hCV-MSC medium or containing 50% supernatant of 1^st^ trimester, term or term PE hCV-MSCs. The supernatant/conditioned medium was collected from hCV-MSCs cultured for 72 h. For direct co-culturing 3x10^4^ hCV-MSCs were seeded into a 0.4 µm pore insert (Greiner bio-one, Frickenhausen, Germany, #657640) with hCV-MSC medium and transferred into six well plates.

Cell surface markers were measured by flow cytometry using a FACSCalibur^TM^ (BD Biosciences, Heidelberg, Germany). After fixation (ice cold 2% PFA, (Sigma-Aldrich, Taufkirchen, Germany, # P6148-1KG) for 15 min at 4°C), cells were washed twice with DPBS (Gibco, Carlsbad, USA, #14190144). The antibodies for staining were purchased from eBioscience (Frankfurt am Main, Germany): FITC-conjugated anti-human CD90 (#11-0909-42), PerCP-Cy5.5-conjugated anti-human CD14 (#45-0149-42), PE-conjugated anti-human CD146 (#12-1469-42), and APC-conjugated anti-human CD31 (#17-0319-42), from BioLegend (Koblenz, Germany): FITC-conjugated anti-human CD34 (#343504), PE-conjugated anti-human CD105 (#323206), or from BD Bioscience: APC‑conjugated anti-human CD106 (#551147), PE-conjugated anti-human CD73 (#550257), FITC-conjugated anti-human CD144 (#560411).

Cell viability was measured via CellTiter-Blue^®^ assay (Promega GmbH, Walldorf, Germany #G808B) as instructed. For cell cycle evaluation, cells were harvested, washed with PBS, fixed with chilled 70% ethanol for 30 min at 4°C and were treated with 1 mg/ml of RNase A (Sigma-Aldrich, Taufkirchen, Germany, #232-646-6) and stained with 100 μg/ml of propidium iodide (PI) (Sigma-Aldrich, Taufkirchen, Germany, #P1304MP) for 30 min at 37°C. DNA content was determined using a FACSCalibur^TM^ (BD Biosciences, Heidelberg, Germany).

**1.2 Isolation of hCV-MSCs**

For isolation of first trimester hCV-MSCs, the placental tissue from the 1^st^ trimester placenta was washed twice with DPBS, and the villous like structures were then collected for further processing. For isolation of term hCV-MSC, the amnion membrane was peeled from term placenta, a 2 cm^3^ villous tissue specimen near the umbilical cord was taken, and minced into small pieces. The following procedures were applied to both 1^st^ trimester and term placenta. The washed tissue was then digested with 15 ml collagenase II 275 U/ml (Worthington, Columbus, USA, #LS004176) and 500 µl Dispase 90 U/ml (Roche, Mannheim, Germany, #4942078001). After 75 min shaking (200 rpm) at 37°C, the enzyme activity was stopped with medium containing 10% FBS (Biochrome, Berlin, Germany) and filtered through a 100 μm mesh (Falcon #352360). The erythrocytes were lysed by red lysis buffer (155 mM NH _4_Cl, 10 mM KHCO_3_, and 0.1 mM EDTA) for 10 min in a 37°C water bath. After twice washing with DPBS (700 g, 5 min), the cells were seeded in a 10 cm dish (Sarstedt, Nümbrecht, Germany, #83.3902) with DMEM (Gibco, Carlsbad, USA, #41966-029) containing 20% FBS (Biochrome, Berlin, Germany), 1% penicillin-streptomycin (Sigma-Aldrich, Taufkirchen, Germany, #P0781) and 1 µg/ml amphotericin B (Sigma-Aldrich, Taufkirchen, Germany, #PHR1662). 5 ng/ml of hFGF (Promega GmbH, Walldorf, Germany, #G5071) was added to the medium of 1^st^ trimester hCV-MSC s until passage 4.

**1.3 Isolation of human umbilical vein endothelial cells (HUVECs)**

HUVEC cells were isolated from human umbilical cord veins by collagenase digestion and cultured. In brief, cells from the umbilical cord lumen were extracted following collagenase I (100 U/mL; Sigma‐ Aldrich, Darmstadt, Germany) digestion by perfusion with Hanks’ Balanced Salt solution (HBSS, Merck, Darmstadt, Germany). The characterization of the isolated HUVECs was performed by staining against CD31 antibody by FACS analysis (data not shown). The purity was around 90-98%. HUVECs were grown in M199 medium (Gibco, Schwerte, Germany) supplemented with 10% fetal bovine serum (Capricorn, Ebsdorfergrund, Germany), large vessel endothelial supplement (Gibco, Carlsbad, USA), penicillin and streptomycin (100 U/mL, Gibco, Carlsbad, USA), and heparin (10 U/mL, Sigma‑Aldrich, Taufkirchen, Germany).

**1.4 Adipogenic, osteogenic and chondrogenic hCV-MSC differentiation**

hCV-MSC differentiation was performed with modifications. hCV-MSCs were cultured with StemPro™ Adipogenesis Differentiation Kit (Thermo Fisher Scientific, Waltham, USA, # A1007001) up to 21 days to induce adipogenic differentiation. The differentiation was conducted with an interval schedule: 3 days in differentiation medium and 4 days in hCV-MSC culture medium for maintenance. After 3 cycles, cells were fixed and stained with DAPI (4’,6-diamidino-2-phenylindole dihydrochloride, Roche, Mannheim, Germany) and analyzed for lipid droplets characteristic of adipocytes, together with a quantification of two important genes *LEPTIN* and *ADIPOQ*. For osteogenic differentiation, hCV-MSCs were incubated with StemMACS OsteoDiff Media (Miltenyi Biotec, Gladbach, Germany, #130-091-678) up to 21 days, fixed, and stained with 2% Alizarin Red S (pH 4.2) to visualize calcific deposition, a hallmark of osteogenic cells. Additionally, important osteogenic differentiation genes *OPN*, *PTCH1* and *KLF4* were evaluated. The chondrogenic differentiation was induced by incubating hCV-MSCs with StemMACS ChondroDiff Media (Miltenyi Biotec, Gladbach, Germany, #130‑091-679) up to 21 days. Cells were fixed and stained with an Alcian blue solution (Merck, Darmstadt, Germany, #TMS-010-C) to visualize sulfated proteoglycans deposits indicative of functional chondrocytes.

**1.5 Immunofluorescence staining of placental tissues and hCV-MSCs**

For immunofluorescence staining, formalin-fixed and paraffin-embedded placental tissue slides were deparaffinized. The slides were incubated in a target retrieval solution for 30 min in a water bath (95°C) for heat-induced epitope retrieval, blocked with peroxidase and incubated with primary antibodies for 1 h at room temperature. The following primary antibodies were used: mouse monoclonal antibody against cytokeratin 7 (DAKO, Frankfurt am Main, Germany, #M7018), rabbit polyclonal antibody against Arl13b (Proteintech, Herford, Germany, #17711-1-1AP) and mouse monoclonal antibody against acetylated α-tubulin (Sigma‑Aldrich, Taufkirchen, Germany, #T6793). This was followed by the incubation with secondary antibodies: rabbit/mouse Cy3 (Abcam, Cambridge, UK, #ab150080, #ab150116) and mouse/rabbit FITC antibody (Abcam, Cambridge, UK, #ab96879, #ab150077) for 30 min at room temperature. Cells on slides were counterstained with DAPI or hematoxylin (Merck, Darmstadt, Germany, #H9627), mounted with Vectashield (Vector Laboratories, Eching, Germany) and analyzed using an AxioObserver.Z1 microscope (Zeiss, Göttingen, Germany) or a confocal laser scanning microscopy (CLSM) using Z-stack images with a HCXPI APO CS 63.0 × 1.4 oil objective (Leica CTR 6500, Heidelberg, Germany).

For the cilium staining, isolated hCV-MSCs were cultured for 2 weeks. Cells above 80% confluence were used for staining and microscopy.

**1.6 Indirect immunofluorescence staining, imaging and signal intensity measurement**

For indirect immunofluorescence staining, cells were seeded on Nunc^TM^ Lab-Tek^TM^ II chamber slides (Thermo Fisher Scientific, Waltham, USA, #C6807-1CS) and fixed with 4% paraformaldehyde containing 0.2% Triton^TM^ X-100 (Sigma-Aldrich, Taufkirchen, Germany, #T8787) for 15 min at room temperature. The following primary antibodies were used: rabbit polyclonal antibody against pericentrin (Abcam, Cambridge, UK, #AB28144), mouse monoclonal antibody against acetylated α-tubulin (Sigma-Aldrich, Taufkirchen, Germany, #T6793), mouse monoclonal antibody against SMO (Santa Cruz Biotechnology, Heidelberg, Germany, #sc-166685), rabbit polyclonal antibody against Arl13b (Proteintech, #17711-1-1AP), mouse monoclonal antibody against vimentin (DAKO, Frankfurt am Main, Germany, #M7020), mouse monoclonal antibody against cytokeratin-7 (DAKO, Frankfurt am Main, Germany, #M7018) and rabbit monoclonal antibody against cytokeratin-18 (Abcam, Cambridge, UK, #ab32118). FITC‐, Cy3‐, and Cy5‐conjugated secondary antibodies were obtained from Jackson Immunoresearch (Cambridgeshire, UK). DNA was stained with DAPI. Slides were examined using an AxioObserver (Zeiss). Z1 microscope and images were taken using an AxioCam MRm camera (Zeiss). The slides were further examined by confocal laser scanning microscopy (CLSM) using Z-stack images with a HCXPI APO CS 63.0 × 1.4 oil objective (Leica CTR 6500, Heidelberg, Germany). A series of Z-stack images were captured at 0.5 μm intervals for overlays (superimposing individual images from confocal Z-sections). Representatives are generated by superimposing (overlay) individual images from confocal Z‑sections.

Fluorescence intensity was analyzed using line-scan-based analysis via ImageJ (National Institutes of Health). The average intensities were measured over a three-pixel-wide line along the axoneme in 10% intervals and normalized against the cilium length by using the ImageJ plugin Plot Roi Profile. The mean values of cilia were obtained for each group within the intervals and were plotted to GraphPad Prism 7 (GraphPad Software Inc., San Diego, USA).

**1.7 Activation of the Hh pathway, zymography and ELISA**

For activating the Hh pathway, hCV-MSCs were first starved in Opti MEM (Gibco, Carlsbad, USA # 11058021) for 24 h, then treated with 400 nM of SAG (Bioscience, Wiesbaden, Germany, #Cay11914-1) in the absence of FCS for 24 h, and stained with indicated antibodies followed by immunofluorescence line-scan-based evaluation.

To investigate the influence of hCV-MSC supernatant on the activity of matrix metallopeptidases (MMPs), HTR or HIPEC cells were cultured for 7 days with medium containing indicated hCV-MSC supernatants (chapter 1.2.). The cells were then incubated in serum free medium for further 24 h and the conditioned medium/supernatant was collected, centrifuged and stored at -80°C for zymography assay. 20 µl of supernatant was incubated with 10 µl sample buffer at room temperature for 15 min and loaded into each well of 8 % SDS gel containing 0.1 % gelatin (Sigma-Aldrich, Taufkirchen, Germany, #G189). After electrophoresis, the gel was soaked with renaturation buffer (2.5% Triton^TM^ X-100), which was changed every 20 minutes for the duration of 1 h. After incubation with developing buffer (50 mM Tris-HCl pH 7.4, 10 mM CaCl_2_, 5 μM ZnCl_2_) at 37°C for 36 h the gel was stained with Coomassie G-250 (40% ethanol, 20% acetic acid) for 1 h and finally de-stained with the buffer contained 10% methanol and 5% acetic acid.

For measuring VEGF-A (DRG Diagnostic, Marburg, Germany, #EIA-4826), β-hCG (DRG Diagnostic, Marburg, Germany, #EIA-1911), and PlGF (RayBiotech Life, Heidelberg, Germany, #P49763), ELISA assays were performed as instructed.

**1.8 RNA extraction and real-time PCR**

Total RNAs of HTR, HIPEC and hCV-MSCs were extracted with EXTRACTME TOTAL RNA KIT (7Bioscience GmbH, Neuenburg am Rhein, Germany). Reverse transcription was performed using GoScript™ Reverse Transcription Mix, Random Primers (Promega GmbH, Walldorf, Germany). Real-time PCR was performed with a StepOnePlus Real-time PCR System (Applied Biosystems). The data were analyzed using StepOne Software v.2.3 (Applied Biosystems). Using the comparative CT method, the gene expression was represented as ΔCT, which is normalized to GAPDH as endogenous control and is inversely related to the amount of target molecules in the reaction. The final results were represented as relative quantification (RQ), indicating the difference in gene expression level between the analyzed samples, by setting the expression value of control condition as 1, in mean with minimum and maximum range. Because the RQ of a group is defined as 2−(ΔCTgroup−ΔCTcontrol), the RQ value for the control group itself leads to the value RQ = 1 without variation.

All probes for gene analysis were obtained from Applied Biosystems: *ADIPOQ* (#00605918_m1), *GAPDH* (#Hs02758991_g1), *GLI1* (#Hs00171790_m1), *IFT88* (#Hs00544051_m1), *KLF4* (#Hs00358836_m1), *LEPTIN* (#Hs00174877), *MMP2* (#Hs01548727_m1), *MMP9* (#Hs00957562_m1) *NANOG* (#Hs04260366_g1), *OPN* (#Hs00959010_m1), *PlGF* (#Hs00903831_g1), *PTCH1* (#Hs00181117_m1), *SMO* (#Hs01090242_m1) and *VEGF* (#Hs00900055_m1).

**1.9 Cellular network formation, cell motility, migration and cell attraction**

Cellular network formation assay was performed as indicated. 96-well plates were coated with 60 µl growth factor reduced matrigel (Cultrex^®^ Basement Membrane Extract, R&D Systems, Minneapolis, United States #3433-005-01) at 37 °C for 1 h. A total of 1 × 10^4^ HUVECs or 2 × 10^4^ HTR cells in medium containing 10% FBS or supernatant of hCV-MSCs were plated on top of pre-solidified matrigel. Cells start to form network like structures once seeded on matrigel. 12 h after incubation, plates were examined with a microscope and images were taken. The resulted images were optimized by inverting the background using Image J (National Institutes of Health, USA). The total number of junctions, master segment length and total number of nodes were quantified using ImageJ plug-in according to the protocols “angiogenesis analyzer from Gilles Carpentier” (Carpentier G. Contribution: angiogenesis analyzer. ImageJ News: ImageJ; 2012).

For motility assay, cells were seeded into 24-well plates with low confluency and were imaged for 12 h at 5 min intervals. All time-lapse imaging was performed with an AxioObserver.Z1 microscope (Zeiss), imaged with an AxioCam MRc camera (Zeiss) equipped with an environmental chamber to maintain proper environmental conditions (37°C, 5% CO_2_). The time-lapse movies were analyzed using ImageJ 1.49i software (National Institutes of Health, USA) with the manual tracking plugin and Chemotaxis and Migration Tool (Ibidi GmbH, Germany). Tracks were derived from raw data points and were plotted in GraphPad Prism 7 (GraphPad software Inc., USA). The accumulated distance was calculated by using the raw data points by the Chemotaxis and Migration Tool. 30 random cells per condition were analyzed and the experiment was repeated independently three times.

Cell migration assays were performed with culture-inserts from Ibidi (Martinsried, Gräfelfing, Germany, #80209). Culture-inserts (cell free gap of 500 µm) were placed in a six cm culture dish and both wells of each insert were filled with cell suspension. HTR (6.5 x 10^4^) and HIPEC (5.5 x 10^4^) were cultured in each well of the culture-inserts. For the direct co-culture, hCV-MSC (2 x 10^5^) were seeded around the ibidi inserts with hCV-MSC medium (Fig. S3A). Culture-inserts were gently removed after at least 8 h and the medium was changed to HTR or HIPEC medium with corresponding conditions. The cells were acquired and imaged at indicated time points with bright-field images. Four pictures of each insert were taken and the experiments were performed in duplicates and at least in three independent times. The open area was measured using the AxioVision SE64 Re. 4.9 software (Zeiss).

The attraction assay measures the length of cell protrusion from the nucleus of hCV-MSCs toward EVTs in a direct co-culture (migration/wound healing assay) using bright-field images with ImageJ 1.49i software (National Institutes of Health, USA). For measurement, cells were fixed and stained for the actin cytoskeleton (phalloidin), phospho-focal adhesion kinase (pFAK) and DNA.

**1.10 Clinical information of human placental tissues as control samples**

Patients with following clinical conditions and undergoing premature birth, whose placental tissues were used for evaluating the cilium length and percentages (10 placental tissue samples from 22-24 weeks, 6 samples from 30-32 weeks of gestational age (GA), used for Fig. 1B and C):

No.1: premature contraction, retroplacental hematoma, premature rupture of membranes (PPROM), GA: 24 weeks+4 days

No.2: prolapsing of amniotic membranes, emergency cerclage, GA: 21+3

No.3: prolapsing of amniotic membranes, emergency cerclage, GA: 21+3

No.4: PPROM, amniotic infection syndrome (AIS), GA: 23+1

No.5: pathologic Doppler, GA: 32+1

No.6: AIS, PPROM, GA: 29+5

No.7: pathologic Doppler, intrauterine growth retardation (IUGR), PPROM, GA: 30+5

No.8: pathologic Doppler, Rhesus incompatibility, intrauterine transfusion (IUT), GA: 30+2

-No.9: initial AIS, PPROM, GA: 31+6

- No.10: pathologic Doppler, GA: 31+6

Patients with following clinical conditions and undergoing premature births, whose placentas were used as early non-PE placental control samples (n = 8, 26-34 gestational weeks, used for Fig. 2A and B):

No. 1: pathologic Doppler, IUGR

No. 2: pathologic Doppler, Rh incompatibility

No.3: pathologic Doppler, maternal kidney failure III, IUGR

No.4: pathologic Doppler, placenta praevia marginalis

No.5: fetal chiari malformation

No.6: pathologic Doppler

No.7: intrahepatic cholestasis of pregnancy

No.8: pathologic Doppler, Lupus, IUGR

**Supplementary table 1:** Clinical information of patients with different gestational age, whose placental tissues were analyzed for cilium size and percentage. Mean value or value range ± standard deviation is shown.

| **Group** | **n** | **Age (years)** | **Gestational age (weeks)** | **BMI** | **GP** | **Birth weight (g)** | **Systolic blood pressure** | **Diastolic blood pressure** | **Proteinuria** | **sFLT / PIGF** |
| --- | --- | --- | --- | --- | --- | --- | --- | --- | --- | --- |
| **22-24 weeks** | 4 | 31  ± 2.45 | 22-24  ± 1.15 | 29.7  ± 5.8 | < 3  ± 0 | 401  ± 73 | 111  ± 7.5 | 76  ± 3.5 | n.d. | n.d. |
| **30-32 weeks** | 6 | 33.5  ± 6.94 | 30-32  ± 0.84 | 23.96  ± 4.48 | < 3 - 34  ± 13.1 | 1545  ± 700 | 114.8  ± 6.7 | 70  ± 8.7 | n.d. | n.d. |
| **38-41 weeks** | 6 | 30.3  ± 4.18 | 38-41  ± 1.94 | 23.52  ± 8.22 | 19 – 85  ± 31.3 | 3442  ± 434 | 121.5  ± 10 | 74.75  ± 5.6 | n.d. | n.d. |

Abbreviation: n.d.: not determined, sFlt: Soluble Fms-like thyrosinkinase-1, PlGF: placental growth factor, GP: growth percentile.

**Supplementary table 2:** Clinical information of early-onset preeclampsia (PE) patients and matched controls, whose placental tissues were analyzed for cilium size and percentage. Mean value or value range ± standard deviation is shown.

| **Group** | **n** | **Age (years)** | **Gestational age (weeks)** | **BMI** | **GP** | **Birth weight (g)** | **Systolic blood pressure** | **Diastolic blood pressure** | **Proteinuria** | **sFLT / PIGF** |
| --- | --- | --- | --- | --- | --- | --- | --- | --- | --- | --- |
| **Non-PE**  **associated** | 8 | 33.9  ± 3.5 | 26 - 34  ± 2.9 | 25.5  ± 4.0 | < 3 - 57  ± 21.6 | 1324  ± 766 | 127  ± 13 | 79  ± 9 | n.d. | n.d. |
| **Early-onset**  **PE** | 12 | 31.8  ± 5.9 | 25 - 32  ± 2.6 | 24.7  ± 4.9 | < 3 - 34  ± 9.6 | 1022  ± 370 | 156  ± 32 | 93  ± 16 | 3235  ± 4570 | 314  ± 107 |
| ***p*-Value** |  | 0.368 | 0.355 | 0.154 | 0.037 | 0.301 | 0.026 | 0.033 | n.d. | n.d. |

Abbreviation: n.d.: not determined, sFlt: Soluble Fms-like thyrosinkinase-1, PlGF: placental growth factor, PE: Preeclampsia, GP: growth percentile.

**Supplementary table 3:** Clinical information of term preeclampsia (PE) patients and matched controls, whose placental tissues were analyzed for cilium size and percentage. Mean value or value range ± standard deviation is shown.

| **Group** | **n** | **Age (years)** | **Gestational age (weeks)** | **BMI** | **GP** | **Birth weight (g)** | **Systolic blood pressure** | **Diastolic blood pressure** | **Proteinuria** | **sFLT / PIGF** |
| --- | --- | --- | --- | --- | --- | --- | --- | --- | --- | --- |
| **Control** | 6 | 38.4  ± 1.52 | 35 - 40  ± 1.94 | 23.52  ± 8.22 | 6 - 30  ± 20.5 | 2692  ± 419 | 121.5  ± 10 | 74.75  ± 5.6 | n.d. | n.d. |
| **Term**  **PE** | 6 | 30.17  ± 3.18 | 37 - 40  ± 1.63 | 23.23  ± 1.48 | < 3 - 24  ±  8.3 | 2288  ± 406 | 166.5  ± 26 | 104.7  ± 22 | 1864  ± 1914 | 273  ± 139 |
| ***p*-Value** |  | 0.47 | 0.44 | 0.47 | 0.14 | 0.066 | 0.006 | 0.017 | n.d. | n.d. |

Abbreviation: n.d.: not determined, sFlt: Soluble Fms-like thyrosinkinase-1, PlGF: placental growth factor, PE: Preeclampsia, GP: growth percentile.

**Supplementary table 4:** Clinical information of term preeclampsia (PE) patients and matched controls, whose placental tissues were collected for the isolation of chorionic mesenchymal stem cells (hCV-MSCs). Mean value or value range ± standard deviation is shown.

| **Group** | **n** | **Age (years)** | **Gestational age (weeks)** | **BMI** | **GP** | **Birth weight (g)** | **Systolic blood pressure** | **Diastolic blood pressure** | **Proteinuria** | **sFLT / PIGF** |
| --- | --- | --- | --- | --- | --- | --- | --- | --- | --- | --- |
| **Control** | 4 | 30.5  ± 3.9 | 40 - 42  ± 0.96 | 21.73± 2.10 | 13 – 86 ± 35.9 | 3273  ± 539 | 123.5  ± 3.42 | 76.5  ± 10.28 | n.d. | n.d. |
| **Term PE** | 3 | 32.67  ± 3.06 | 37 - 40  ± 1.53 | 25.8 ± 5.09 | 3 – 64 ± 29.7 | 2573 ± 595 | 171  ± 6.56 | 108.3  ± 6.66 | 4420  ± 3465 | 310.33 ± 118.89 |
| ***p*-Value** |  | 0.355 | 0.009 | 0.135 | 0.151 | 0.039 | 0.0004 | 0.0028 | n.d. | n.d. |

Abbreviation: n.d.: not determined, sFlt: Soluble Fms-like thyrosinkinase-1, PlGF: placental growth factor, PE: Preeclampsia, GP: growth percentile.

**
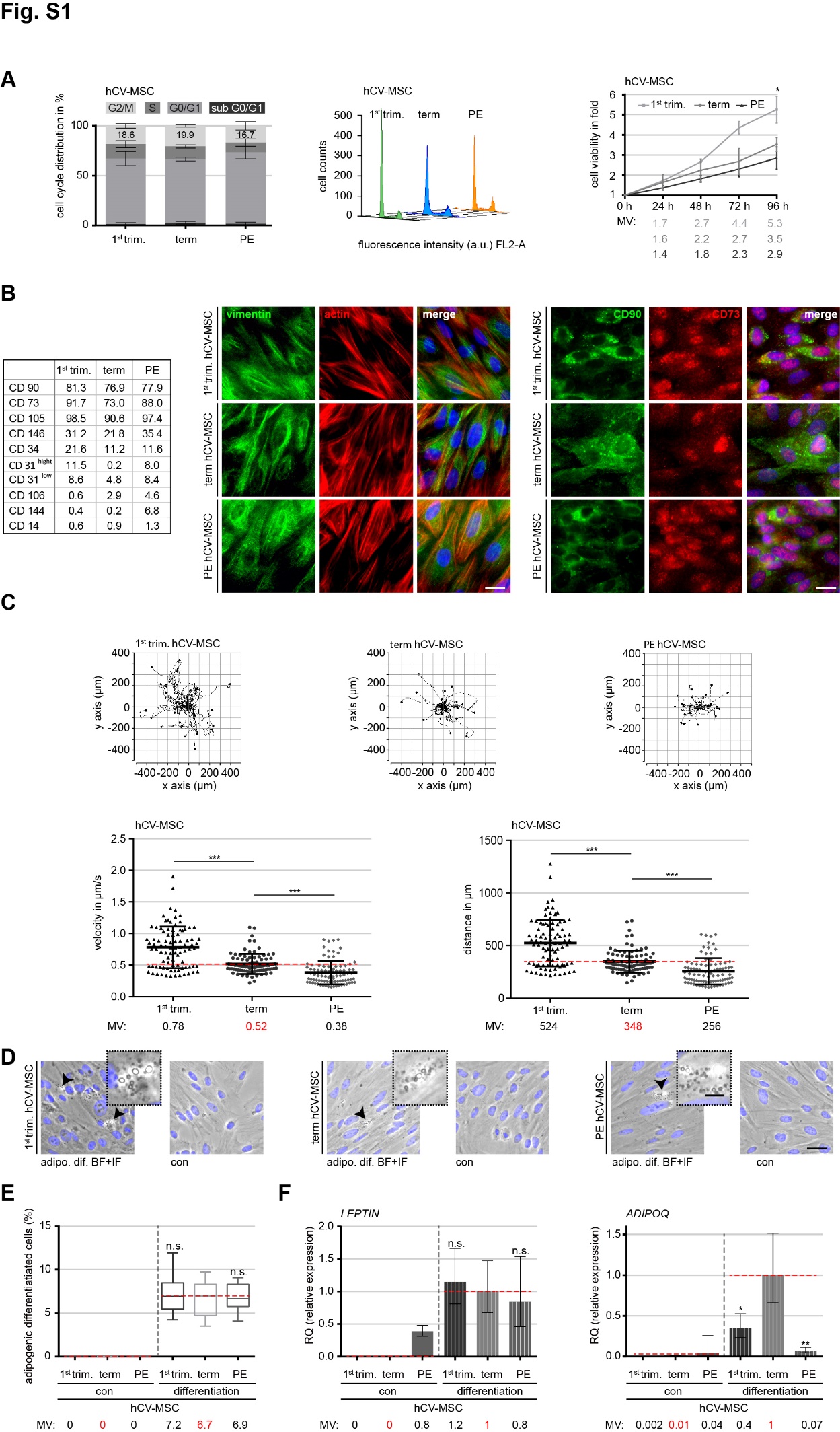
**

**Figure S1: hCV-MSCs from 1^st^ trimester, term and PE placentas display comparable cell surface maker profiles, proliferation, and differentiation capacity.**

(A, left graph) Cell cycle distribution was analyzed using a FACSCalibur^TM^. The cell cycle phases of hCV-MSCs are presented in percentage and the results were derived from three independent experiments. (A, middle graph) Representative FL2-A histogram profiles of the cell cycle are shown. (A, right graph) hCV-MSCs were seeded in 96-well plates for 0, 24, 48, 72 and 96 h. Cell viability was measured via CellTiter-Blue^®^ assay. The results are from three independent experiments and presented as mean ± SEM. **p* < 0.05. (B) Flow cytometric analyses of positive cell surface markers CD90, CD73, CD105 and CD146, and negative markers CD14, CD31^low/high^, CD34, CD106, CD144 (B, left table). Representative staining of hCV-MSCs are shown for vimentin, actin and DNA (B, middle graph) or cell surface markers CD90 and CD73 (B, right graph). Scale: 20 μm. (C) Time-lapse microscopy was performed with hCV-MSCs for up to 12 h. Random motility of these cells was analyzed (n = 90 cells for each group). Representative trajectories of individual cells are shown (C, upper panels). Evaluated accumulated velocity (C, lower left plot) and distance (C, lower right plot) from three independent experiments are shown. Unpaired Mann–Whitney *U*-test, *** *p* < 0.001. (D-F) hCV-MSCs from 1^st^ trimester, term and term PE placentas were subjected to adipogenic differentiation for 21 days. (D) Representative images for adipocytes are shown. Scale: 40 μm. Insert scale: 10 μm (arrowheads depict lipid vacuoles). (E) The percentage of differentiated adipocytes was evaluated by counting cells with lipid vacuoles. The quantification of cells displaying lipid vacuoles is shown as bar graph with mean ± SEM (n = 15, pooled from three independent experiments with three individual hCV-MSCs). (F) Expression levels of two adipogenic differentiation related genes *LEPTIN* (E, left graph), *ADIPOQ* (E, right graph) in control and hCV-MSCs upon differentiation. The results are from three independent experiments and presented as RQ with minimum and maximum range. Student’s t-test was used. ∗p < 0.05, ∗∗p < 0.01.

**
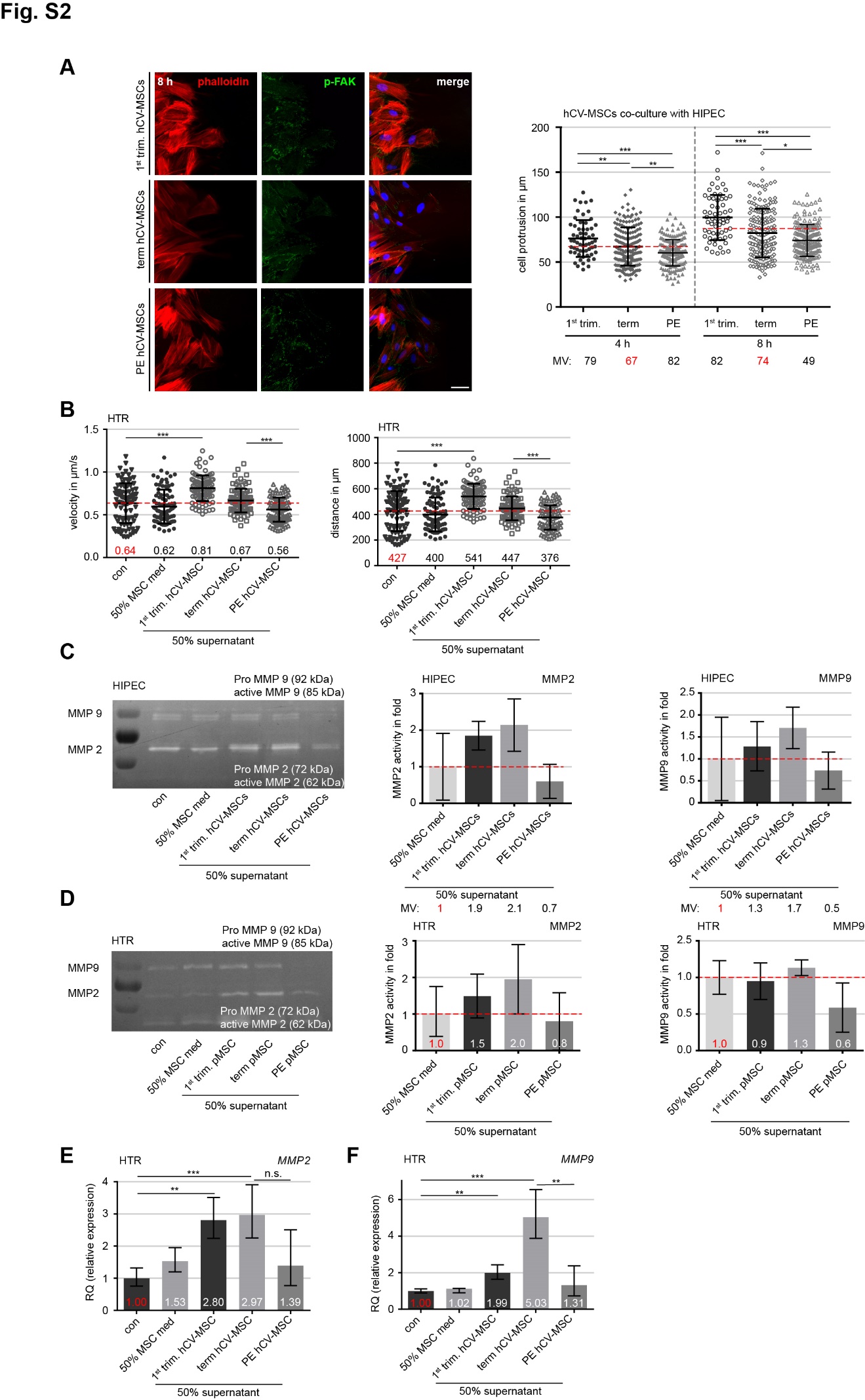
**

**Figure S2: The motility and homing ability is impaired in PE hCV-MSCs as well as their capacity to stimulate motility and MMP2/9 activity in EVT cells.**

(A) HTR cells and hCV-MSCs from 1^st^ trimester, term and term PE placentas were seeded into each Ibidi chamber. After 8 h, the chambers were removed and the hCV-MSCs started to migrate toward HTR cells. After 4 and 8 h bright-field images were taken for analysis. For fluorescence visualization, the cells were stained with phalloidin (actin filaments, red), p-FAK (focal adhesion marker, green) and DNA (DAPI, blue). (A, left panel) Representatives of the hCV-MSCs at the migrating front after 8 h are shown. Scale: 50 μm. (A, right graph) The length of the cellular protrusions of hCV-MSCs toward HTR cells was quantified, and was presented as scatter plot showing mean ± SEM (n = 180 protrusions, pooled from three independent experiments. (B) Single HTR cells were tracked after the treatment with indicated medium (control medium or medium containing 50% supernatant from hCV-MSCs of 1^st^ trimester, term or term PE placentas) using time-lapse microscopy to analyze their cell motility. The velocity (C, left plot) and accumulated distance (C, right plot) were evaluated for each individual treatment. The results from three experiments are depicted as scatter plots showing mean ± SEM (n = 90 cells). Unpaired Mann-Whitney *U* test was used for (A and B). ∗p < 0.05, ∗∗p < 0.01, ∗∗∗p < 0.001. (C and D) HIPEC (C) and HTR (D) cells were incubated with indicated medium (control medium or medium containing 50% supernatant from hCV-MSCs of 1^st^ trimester, term or term PE placentas) for 7 days. Afterwards, cells were starved-cultured for 24 h and the supernatants were collected for zymography assay to measure the activity of MMP2 and MMP9. (C and D, left panel) Representatives indicate the activity of MMP2 (lower band) and MMP9 (upper band). (C and D, middle and right graph) Quantification of MMP2 and MMP9 activity, normalized to the MSC control medium, is shown. The results are from three independent experiments and presented as bar graphs with mean ± SEM. (E) Total RNAs were also extracted from treated HTR cells for analyzing gene levels of *MMP9* (E) and *MMP2* (F). The data are based on three independent experiments and presented as RQ with minimum and maximum range and statistically analyzed. RQ: relative quantification of the gene expression. Student’s t-test was used. ** p < 0.01, *** p < 0.001.

**
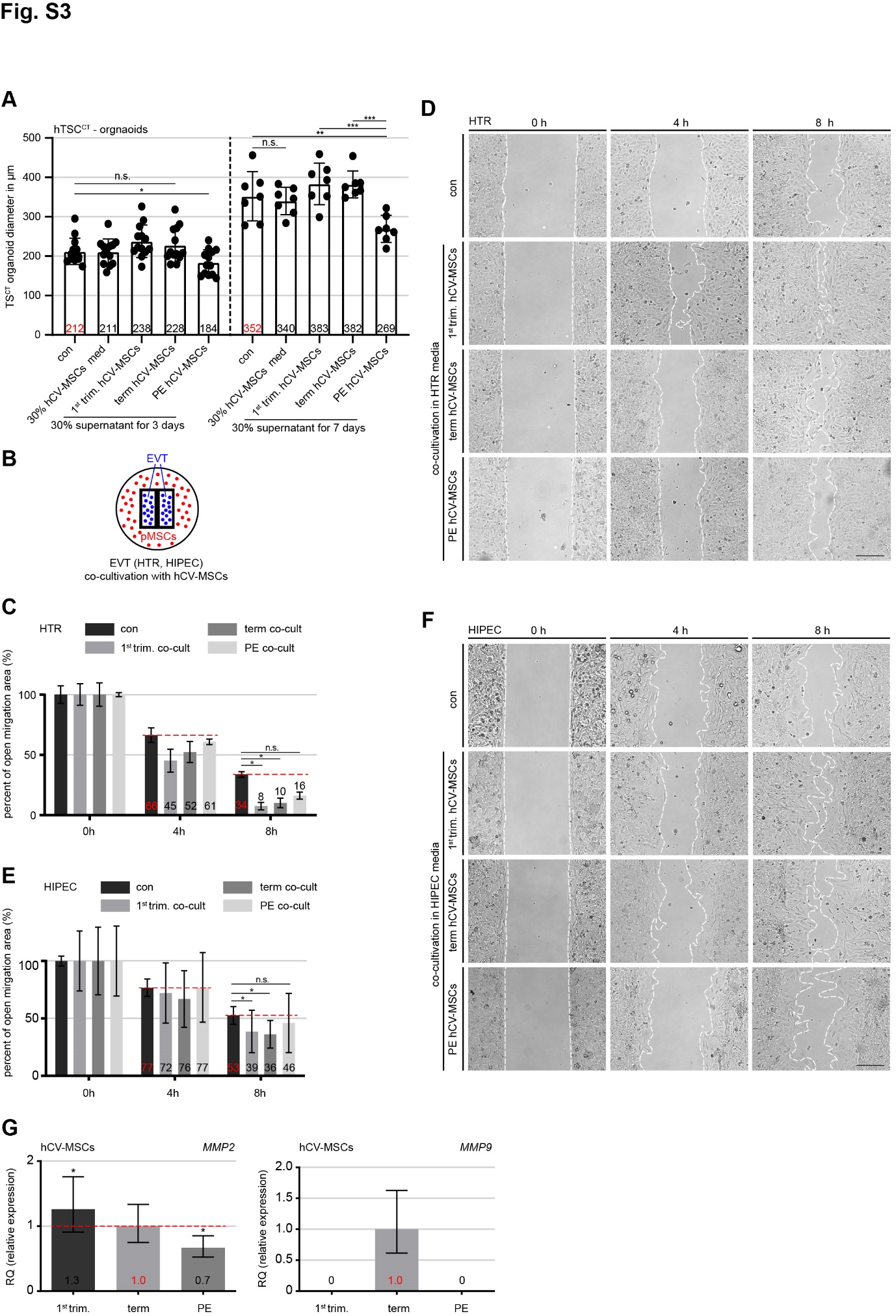
**

**Figure S3: hCV-MSCs from term PE placentas reduced their ability to support growth of placental organoids and migration of EVT cells.**

(A) Placental organoids were formed for 72 h by using hTSC^CT^ cells and treated then for up to 7 days with 30% supernatant from 1^st^ trimester, term control or term PE hCV-MSCs. The organoids were stained against β-hCG (red), pHH3 (green) and DNA (DAPI, blue), and their diameters were microscopically evaluated. The results are presented as scatter graphs showing the mean ± SEM (n = 7-10 organoids, from three different hCV-MSCs supernatants for each group). (B-F) Illustration of cell co-culture experiment. HIPEC/HTR cells (blue) were seeded into Ibidi chambers and surrounded by indicated hCV-MSCs from 1^st^ trimester, term control and term PE placentas (red). After 8 h the chambers were removed, and the medium changed to HTR or HIPEC medium. Images were taken at indicated time points (0, 4, 8 h) to document the migration font. (B and D) Quantification of the open area between both migration fronts at various time points, for HTR (B) and HIPEC cells (D). The cell-free area at 0 h was assigned as 100%. The results from three independent experiments are presented as mean ± SEM. Unpaired Mann–Whitney *U*-test was used. *p < 0.05, **p < 0.01. (C and E) Representatives of the migration front are shown. White dashed line depicts the free area of the migration front. Scale: 200 µm. (G) Total RNAs were extracted from hCV-MSCs for analyzing gene levels of *MMP2* (G, left graph) and *MMP9* (G, right graph). The data are based on three independent experiments and presented as RQ with minimum and maximum range. RQ: relative quantification of the gene expression. Student’s t-test was used. *p < 0.05.


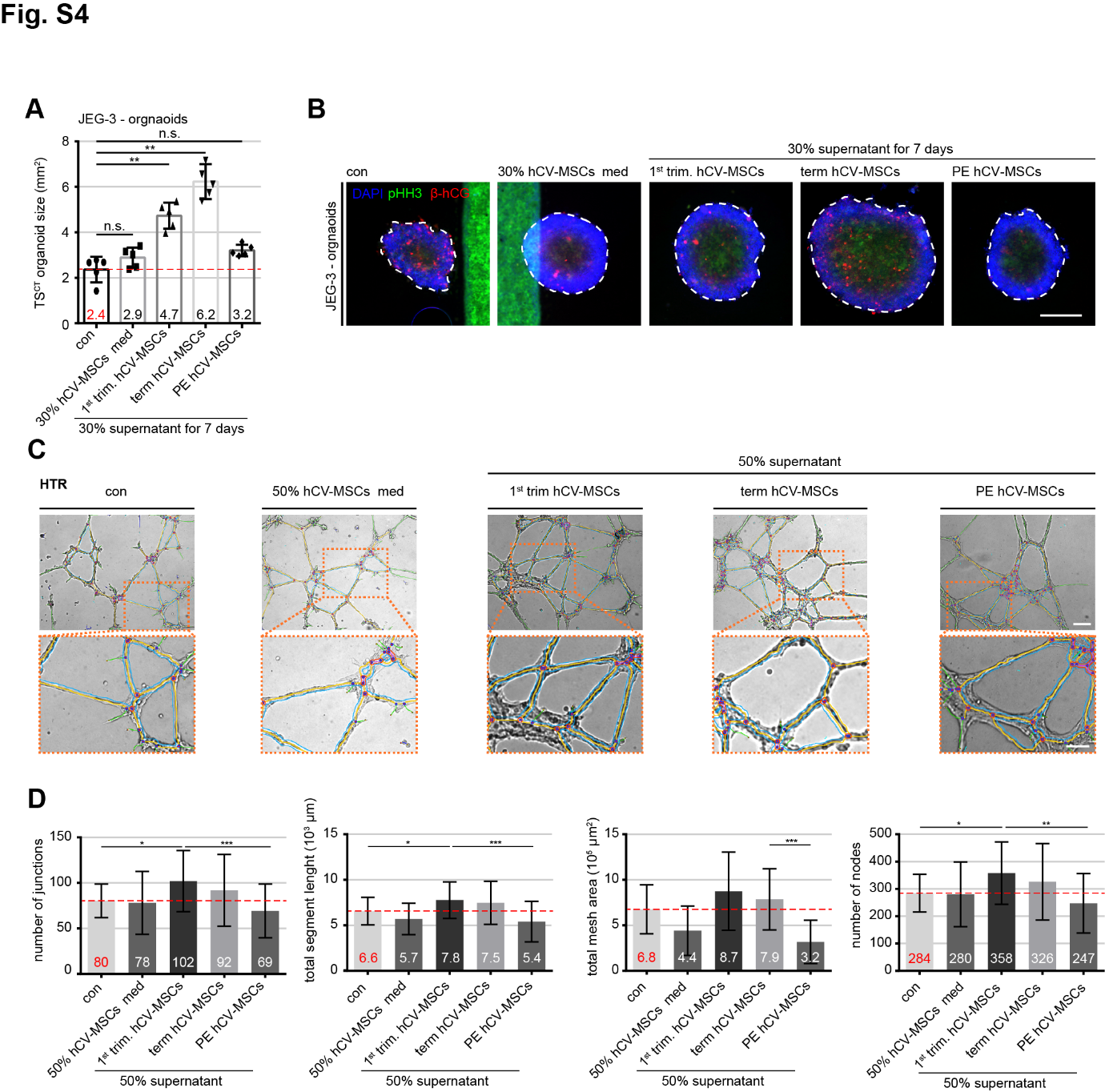


**Figure S4: PE hCV-MSCs are less capable of supporting proliferation of JEG-3 organoids and network formation of HTR cells.**

(A and B) Placental organoids were generated for 96 h by using JEG-3 cells and treated then for up to 7 days with 30% supernatants from 1^st^ trimester, term or PE hCV-MSCs. The organoids were stained against β-hCG (red), pHH3 (green) and DNA (DAPI, blue) and microscopically evaluated. The results of the organoid area are presented as scatter graphs showing the mean ± SEM (n = 5 organoids, from three different hCV-MSC supernatants for each group) (B). Representative images of stained JEG-3 organoids treated with different supernatants for 7 days are shown (white dotted lines indicate measured areas). Scale: 350 μm. Student’s t-test was used. ** p < 0.01. (C and D) Cellular network formation assay was performed with HTR cells cultured with different medium as indicated (control medium, control medium containing 50% MSC normal medium, or containing 50% supernatants from hCV-MSCs of 1st trimester, term or term PE placentas). (C) Representatives of light microscopic images are shown (green: branches; cyan: twigs; yellow: master segments; red surrounded by blue: nodes surrounded by junctions; blue surrounded by red: master junctions). Scale: 200 μm. (D) Quantification of total number of junctions (D, 1st graph), the total segment length (D, 2nd graph), total mesh area (D, 3rd graph) and total number of nodes (D, 4th graph) is shown. The results are based on three independent experiments (n = 15 pictures of each condition per group) and presented as bar graphs with mean ± SEM. Student’s t-test was used. *p < 0.05, ** p < 0.01, *** p < 0.001.
